# Niosomal Mexiletine Attenuates Rotenone-Induced Parkinsonism via Modulation of the circDLGAP4/miR-134-5p/p-CREB Axis and Restoration of Mitochondrial–Autophagic Homeostasis

**DOI:** 10.3390/ph19101654

**Published:** 2026-10-20

**Authors:** Eman M. Raafat, Fares Farhan Alanazi, Sarah Khalifah Alanazi, Yara E. Mansour, Mohammed S. Teiama, Shimaa K. Mohamed

**Affiliations:** 1 Department of Pharmacology and Toxicology, Faculty of Pharmacy, Capital University, Ein Helwan, Cairo 11795, Egypt; eman.raffat@pharm.capu.edu.eg; 2 Maternity and Children Hospital, Arar 91431, Saudi Arabia; ifares1992@gmail.com; 3 Department of Chemistry, Faculty of Science, University of Hail, Hail 81411, Saudi Arabia; sarah1994alenzi@gmail.com; 4 Department of Pharmaceutical Chemistry and Pharmacognosy, College of Pharmacy, Qassim University, Buraydah 51452, Saudi Arabia; 5 Department of Pharmaceutics and Industrial Pharmacy, Faculty of Pharmacy, Capital University, Ein Helwan, Cairo 11795, Egypt; dr_mteiama84@pharm.capu.edu.eg; 6 Department of Pharmaceutics and Industrial Pharmacy, Faculty of Pharmacy, Galala University, Attaka, Suez 43713, Egypt

**Keywords:** rotenone, niosomal mexil, mitochondrial dysfunction, circDLGAP4/miR-134/p-CREB, PGC-1α, autophagy

## Abstract

**Background**: Parkinson’s disease (PD) is distinguished by progressive dopaminergic degeneration driven by the convergence of oxidative stress, neuroinflammation, mitochondrial dysfunction, and impaired autophagy. Mexiletine (Mexil), which has demonstrated antioxidant properties, has not been systematically investigated yet for its potential to mitigate mitochondrial-dysfunction-related neurodegeneration in PD. Using an integrated computational and experimental approach, we characterized, for the first time, the neuroprotective mechanisms of Mexil’s action against rotenone (Rot)-induced Parkinsonism and employed niosomes as a nanocarrier platform to enhance mexil delivery and augment its neuroprotective potential in a Parkinson’s disease model. **Methods**: Molecular docking identified candidate molecular targets underlying niosomal Mexil’s neuroprotective potential. Sprague–Dawley male rats were assigned to control, Rot, and Rot plus niosomal Mexil (30 and 50 mg/kg) groups. Motor performance was evaluated, while dopaminergic degeneration, oxidative stress, neuroinflammation, mitochondrial dysfunction, and autophagy were assessed biochemically, histopathologically, and immunohistochemically. **Results**: Docking revealed promising interactions between Mexil and both phosphorylated cAMP response element-binding protein (p-CREB) and alpha-synuclein, with binding scores of −5.2 and −4.9 kcal/mol, respectively. Niosomal Mexil improved motor function, restored dopamine and tyrosine hydroxylase levels, and curtailed alpha-synuclein accumulation. Most notably, niosomal Mexil engaged a previously unexplored circular RNA DLGAP4/microRNA-134-5p/p-CREB axis, promoted mitochondrial biogenesis via the phosphorylated AMP-activated protein kinase (p-AMPK)/peroxisome proliferator-activated receptor gamma coactivator 1-alpha (PGC-1α)/sirtuin-1 (SIRT-1) pathway, and rebalanced mitochondrial dynamics. These changes were accompanied by restored autophagic axis and suppressed oxidative stress and inflammation. **Conclusions**: Niosomal Mexil confers neuroprotection against Rot-induced Parkinsonism by orchestrating oxidative, mitochondrial, autophagic, and inflammatory pathways, positioning it as a mechanistically novel disease-modifying candidate warranting further preclinical evaluation in PD.

## 1. Introduction

Parkinson’s disease (PD) is a progressive neurodegenerative disorder characterized by the selective loss of substantia nigra dopaminergic neurons and subsequent striatal dopamine depletion, resulting in cardinal motor symptoms including resting tremor, rigidity, bradykinesia, and postural instability. Pathologically, PD is characterized by the accumulation of Lewy bodies enriched in ubiquitin and α-synuclein [1].

The etiology and pathogenesis of Parkinson’s disease (PD) are poorly understood. Genetic factors may account for some cases of PD, but most cases are sporadic and arise from an intricate interaction between genetic predisposition and environmental exposures [2,3]. Fundamental mechanisms implicated in the initiation and progression of PD are oxidative stress, neuroinflammation, and mitochondrial dysfunction [2]. Despite progress in conventional therapies, most are symptomatic, alleviating clinical manifestations but not effectively modifying or halting disease progression [4].

It has been demonstrated that noncoding RNAs (ncRNAs) are involved in various physiological and pathological processes. Circular RNAs (circRNAs) are a class of ncRNAs engendered by back-splicing, which are considered stable and evolutionarily conserved regulators of gene expression through miRNA sponging, transcriptional regulation, protein interaction, and translation of peptides. Encouraging evidence implies that circRNAs participate in the pathogenesis of PD via regulating α-synuclein, oxidative stress, apoptosis, and autophagy [5].

Interestingly, circRNA DLG associated protein 4 (circDLGAP4) is downregulated in PD models, and circDLGAP4 deficiency promotes mitochondrial dysfunction, impaired autophagy, and apoptosis, while circDLGAP4 overexpression exerts neuroprotective effects [6,7]. Mechanically, circDLGAP4 acts as a sponge of miR-134-5p, which relieves the suppression of cAMP response element-binding protein (CREB) by miR-134-5p and activates downstream targets including B-cell lymphoma 2 (Bcl-2), peroxisome proliferator-activated receptor gamma coactivator 1-alpha (PGC-1α), and brain-derived neurotrophic factor (BDNF) [6,8]. Thus, the circDLGAP4/miR-134-5p/CREB axis might be an important regulatory pathway in PD pathogenesis.

Mitochondria control substrate metabolism, ATP generation, and cell-death signaling to preserve tissue and cellular homeostasis. Mitochondrial dynamics, biosynthesis, and mitophagy sustain their functional integrity and abundance [9,10]. While physiological fission maintains mitochondrial number and intracellular distribution, excessive fission impairs axonal transport, increases oxidative stress, upsurges mitochondrial heterogeneity, and destabilizes the mitochondrial network [10]. Mitochondrial biogenesis, however, creates new mitochondria in the presence of increased energy demand [11]. The disruption of these regulatory processes is a major contributor to sporadic and familial Parkinson’s disease, promoting excessive ROS production, mtDNA depletion or deletion, respiratory chain impairment, ATP deficiency, and loss of nigrostriatal integrity [12,13]. Dopaminergic neurons of the substantia nigra pars compacta have high metabolic and energy demands, which make them particularly vulnerable to mitochondrial injury and gradual degeneration [14].

Autophagy is a critical regulator of neuronal homeostasis, orchestrating the clearance of damaged organelles and aggregation-prone proteins, such as dysfunctional mitochondria and toxic α-synuclein species [15]. Thus, disruption of this degradative pathway could contribute to PD through accumulation of mitochondrial damage and proteotoxic stress. In addition to cellular quality control, autophagy is involved in energy metabolism by recycling macromolecules into substrates for ATP production, especially under metabolic stress [16]. In this way, impaired autophagic processes can worsen bioenergetic dysfunction through the combined effects of reduced mitochondrial clearance and reduced substrate availability, thereby initiating a feed-forward cycle that increases the vulnerability of dopaminergic neurons [17].

Mexiletine (Mexil) is a drug used to suppress pathological neuronal hyperexcitability, but its central therapeutic efficacy is limited by suboptimal brain delivery [18]. Niosomes are biocompatible nanocarriers consisting of non-ionic surfactants and cholesterol, which can encapsulate hydrophilic and hydrophobic medicines. Their membrane-like structure promotes drug stability and bioavailability and may enable tailored distribution across the blood–brain barrier with fewer systemic adverse effects [19]. Therefore, niosome-based delivery represents a rational strategy for enhancing the cerebral bioavailability and neuroprotective efficacy of mexiletine.

Mexil is a class IB antiarrhythmic drug used to treat ventricular arrhythmias. It acts mainly by blocking voltage-gated sodium channels in a use-dependent manner, thereby increasing the threshold for depolarization and limiting abnormal cellular excitability [20]. In addition to its cardiac effects, Mexil has shown antioxidant and neuroprotective activity in experimental models of cerebral ischemia, spinal cord injury, and traumatic brain injury [20,21]. Systemically administered Mexil has also been reported to preserve neural tissue and reduce demyelinating damage [22].

Neurodegenerative diseases represent a major global therapeutic challenge, primarily due to the stringent selectivity of the blood–brain barrier (BBB), which severely restricts the central nervous system bioavailability of conventional pharmaceuticals [23,24]. To overcome these physiological transport barriers, niosomes—microscopic, synthetic nanovesicles defined by a self-assembled closed bilayer matrix composed primarily of non-ionic surfactants and cholesterol—have emerged as a highly versatile drug delivery platform [24,25]. In these systems, the amphiphilic non-ionic surfactants drive spontaneous membrane assembly, while cholesterol optimizes membrane fluidity, rigidity, and overall physical stability [24,25]. Due to their unique core–shell architecture, niosomal formulations exhibit a dual-encapsulation capability that accommodates hydrophilic therapeutic agents within their central aqueous core and lipophilic compounds inside of the hydrophobic bilayer, effectively shielding loaded drugs from enzymatic degradation and extending systemic circulation time [25]. Furthermore, functionalizing niosomal surfaces with targeting ligands or utilizing non-invasive routes facilitates targeted central nervous system accumulation while minimizing off-target peripheral toxicity [23,25]. Consequently, niosomes represent a highly promising, stable, and cost-effective nanomedicine strategy to restore therapeutic drug concentrations in damaged neurodegenerative tissues [22,23].

Mexil’s potential role in PD has received little attention. Thus, the present study aimed to examine the neuroprotective effects of niosomal Mexil in a rotenone-induced rat model of Parkinsonian neurodegeneration with a comprehensive evaluation of behavioral, dopaminergic, mitochondrial, autophagic, oxidative, inflammatory, apoptotic, and energy-related outcomes associated with the circDLGAP4/miR-134-5p/CREB and AMPK/SIRT1/PGC-1α pathways to elucidate the possible mechanisms behind the observed neuroprotective effects and provide a rationale for future preclinical studies.

## 2. Results

### 2.1. Fourier Transform Infrared (FTIR)

**Figure 1:**
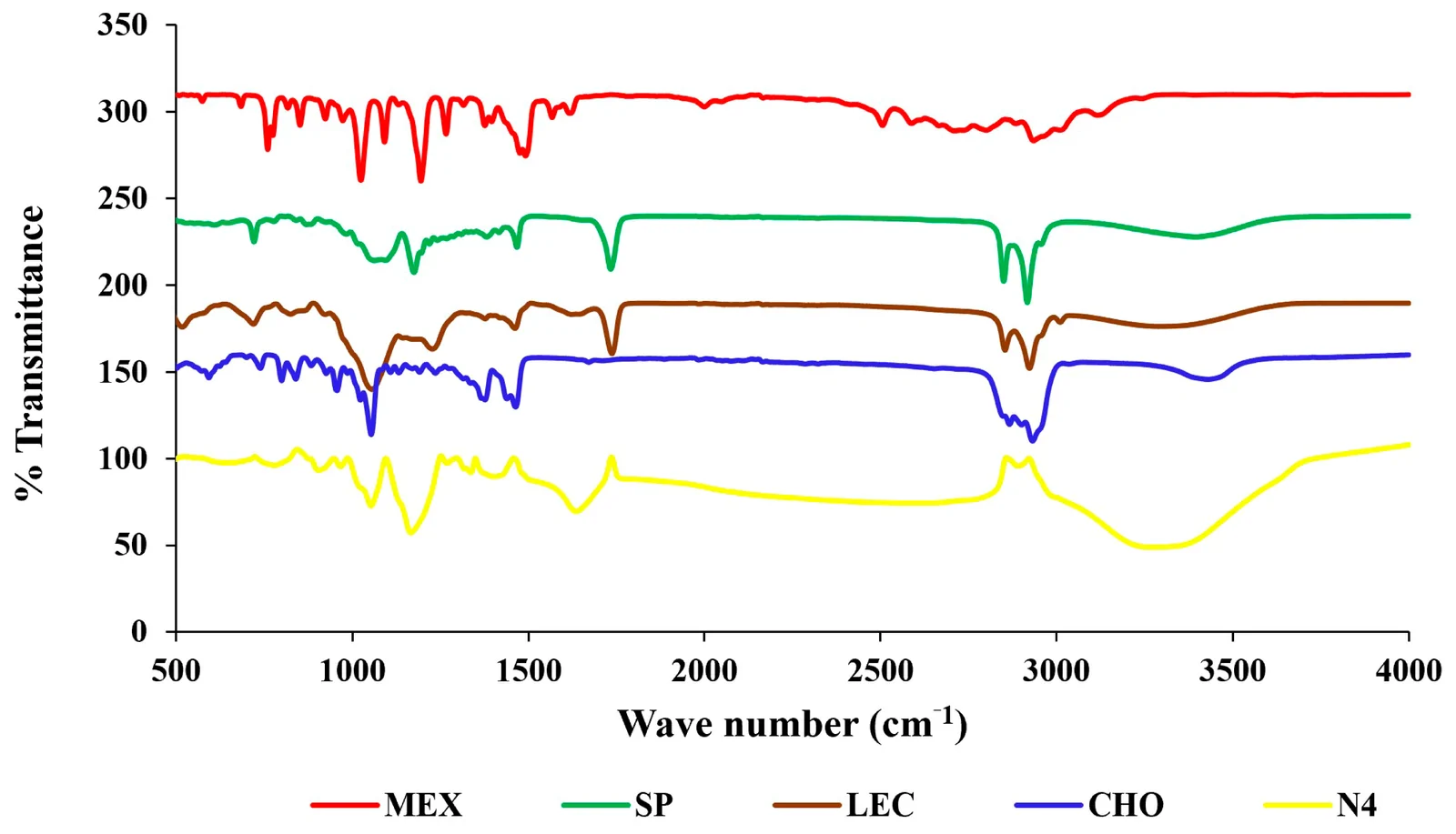
FTIR spectrum of mexiletine hydrochloride, cholesterol, lecithin, Span 60, optimized Mexil niosomes (N4).

FTIR analysis confirmed the characteristic functional groups of Mexil HCl and niosomal excipients (Figure 1). Mexil HCl exhibited characteristic bands at 3293 cm^−1^ (N-H), 2858 and 2514 cm^−1^ (aliphatic C-H), 1638 cm^−1^ (aromatic C=C), 1252 cm^−1^ (C-O), and 1171 cm^−1^ (C-N). In Mexil-loaded niosomes, the attenuation and slight shifting of the characteristic bands of Mexil and excipients, particularly in the region of 3200–3000, 2920, and 1740 cm^−1^, indicated hydrogen bonding and hydrophobic interactions in the vesicular bilayer. The absence of new peaks and major structural changes indicated good compatibility of Mexil and excipients without any chemical degradation, which further supported the successful incorporation of Mexil into the niosomal system.

### 2.2. Entrapment Efficiency (EE%)

**Figure 2:**
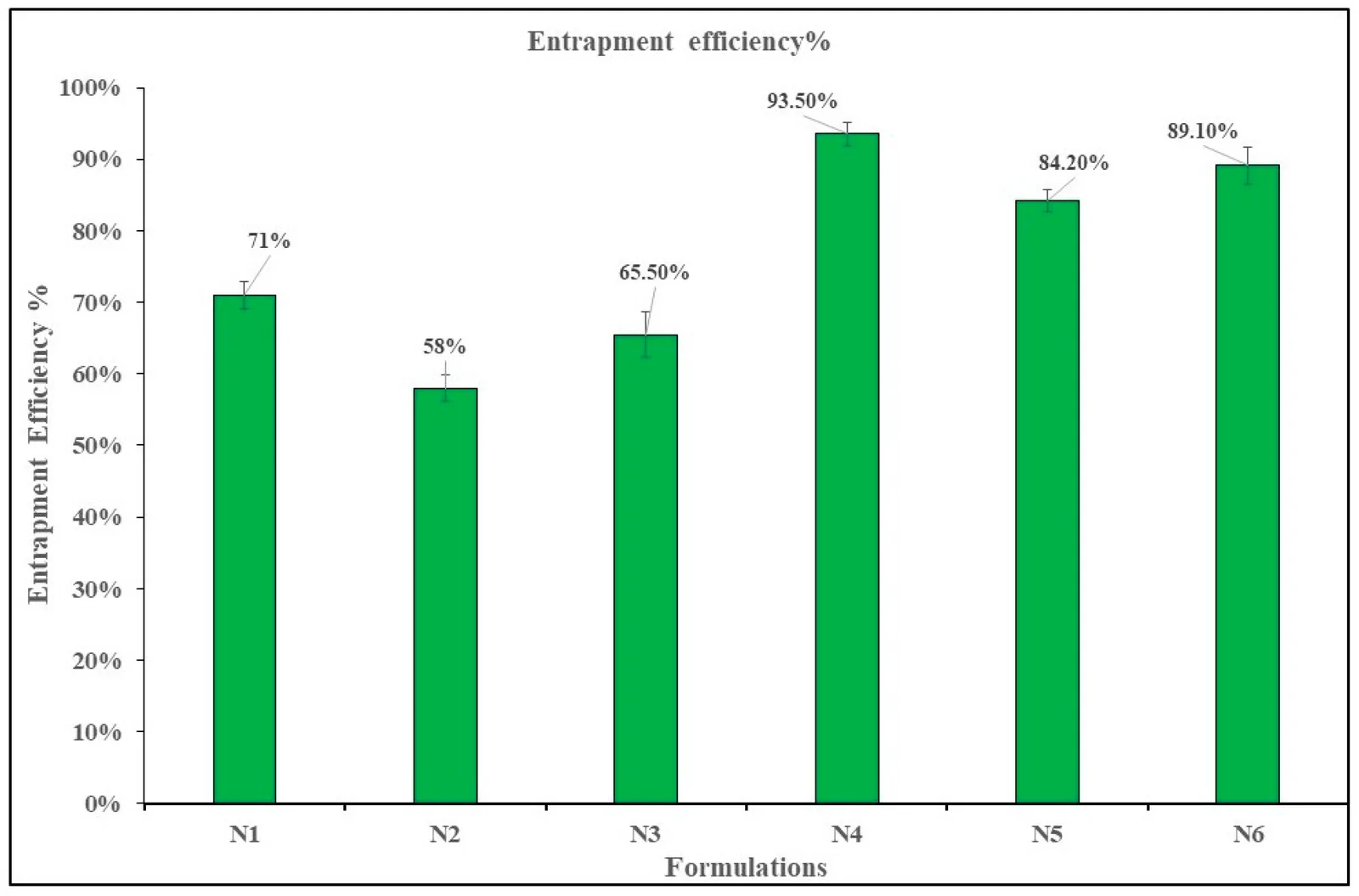
Entrapment efficiency % in different formulations (N1–N6). Measures are summarized as mean ± SD (*n* = 3).

The %EE of Mexil-loaded niosomes was found to be in the range of 58.0 ± 1.8% to 93.0 ± 1.7% (Figure 2). Formulations prepared with higher lecithin content (225 mg; N4–N6) were found to have significantly higher EE% (84.2–93.0%) than those with 150 mg of lecithin (58.0–71.0%). This increase shows the importance of phospholipid concentration in the improvement of the drug retention in the lipid bilayer. This agrees with previous studies where it was observed that an increase in lipid concentration leads to an increase in the hydrophobic core and drug entrapment [26]. Among all of the formulations, N4 showed the highest EE% (93.0 ± 1.7%), followed by N6 (89.1 ± 2.6%) and N5 (84.2 ± 1.5%). This good encapsulation can be explained by the synergism of high levels of lecithin, the optimal Span 60/cholesterol ratio, and the rigid and low-permeability bilayer properties of Span 60 [27,28]. Span 60 has a long-saturated alkyl chain (C16) and high phase-transition temperature (Tc ≈ 53 °C), which increases tight molecular packing and decreases vesicle leakiness [28,29]. So, N4 was identified as the optimal formulation for Mexil entrapment.

### 2.3. Particle Size, Zeta Potential, and Polydispersity Index (PDI)

**Figure 3:**
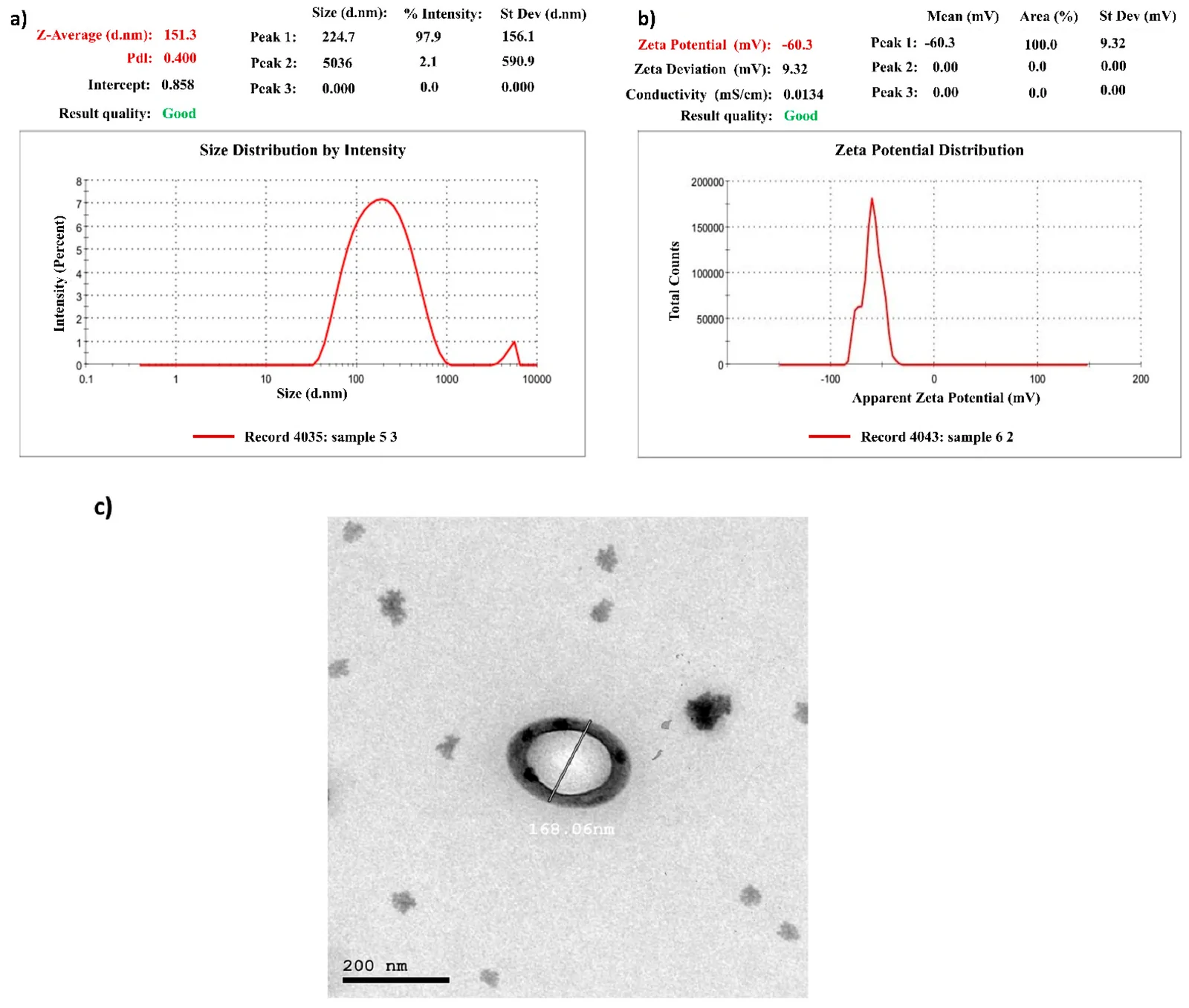
Illustrative chart of (**a**) particle size, (**b**) zeta potential values (ZP) of the prepared niosomal dispersions, and (**c**) Representative Transmission electron microscopy (TEM) image of the optimized niosomal formulation (N4).

The particle sizes of the prepared Mexil-loaded niosomes were in the range of 150.4 ± 4.8 nm to 225.7 ± 3.5 nm, confirming the formation of nanosized vesicles. Formulations N1–N3 showed smaller vesicle sizes (150.4–153.5 nm), while increasing the lecithin content in N4–N6 caused an increase in vesicle size (170.5–225.7 nm), reflecting the increased bilayer content and vesicle size. All formulations showed negative zeta potential values ranging from −34.5 ± 5.29 mV to −64.0 ± 8.08 mV, indicating adequate electrostatic repulsion and good colloidal stability. The higher negative values observed for N4–N6 could be associated with their higher lecithin content and consequent bilayer organization. The PDI values ranged from 0.393 ± 0.02 to 0.956 ± 0.10, reflecting different size distributions of different formulations. N4 showed the lowest PDI (0.393 ± 0.02), indicating the most uniform vesicle population, whereas N5 showed the highest PDI (0.956 ± 0.10), indicating significant size heterogeneity. Based on the overall physicochemical features, N4 was selected as the optimized formulation, being characterized by its high EE% (93.0 ± 1.7%), nanoscale particle size (205.6 ± 3.9 nm), highly negative zeta potential (−60.3 ± 8.8 mV), and lowest PDI (0.393 ± 0.02). These features suggest a good compromise between drug entrapment, vesicle size, colloidal stability, and size uniformity Figure 3a,b.

The optimized niosomes were characterized through a TEM study, which revealed the spherical to near spherical, well-defined and distinct vesicular structures with minimum aggregation. The vesicle size obtained from the TEM micrographs ranged from 168 to 236 nm, which is in reasonable agreement with the hydrodynamic size reported by dynamic light scattering (DLS). The TEM results confirmed the nano-size and well-defined morphology of the optimized niosomal formulation (Figure 3c).

### 2.4. In Vitro Mexiletine Release from Niosomal Dispersions

**Figure 4:**
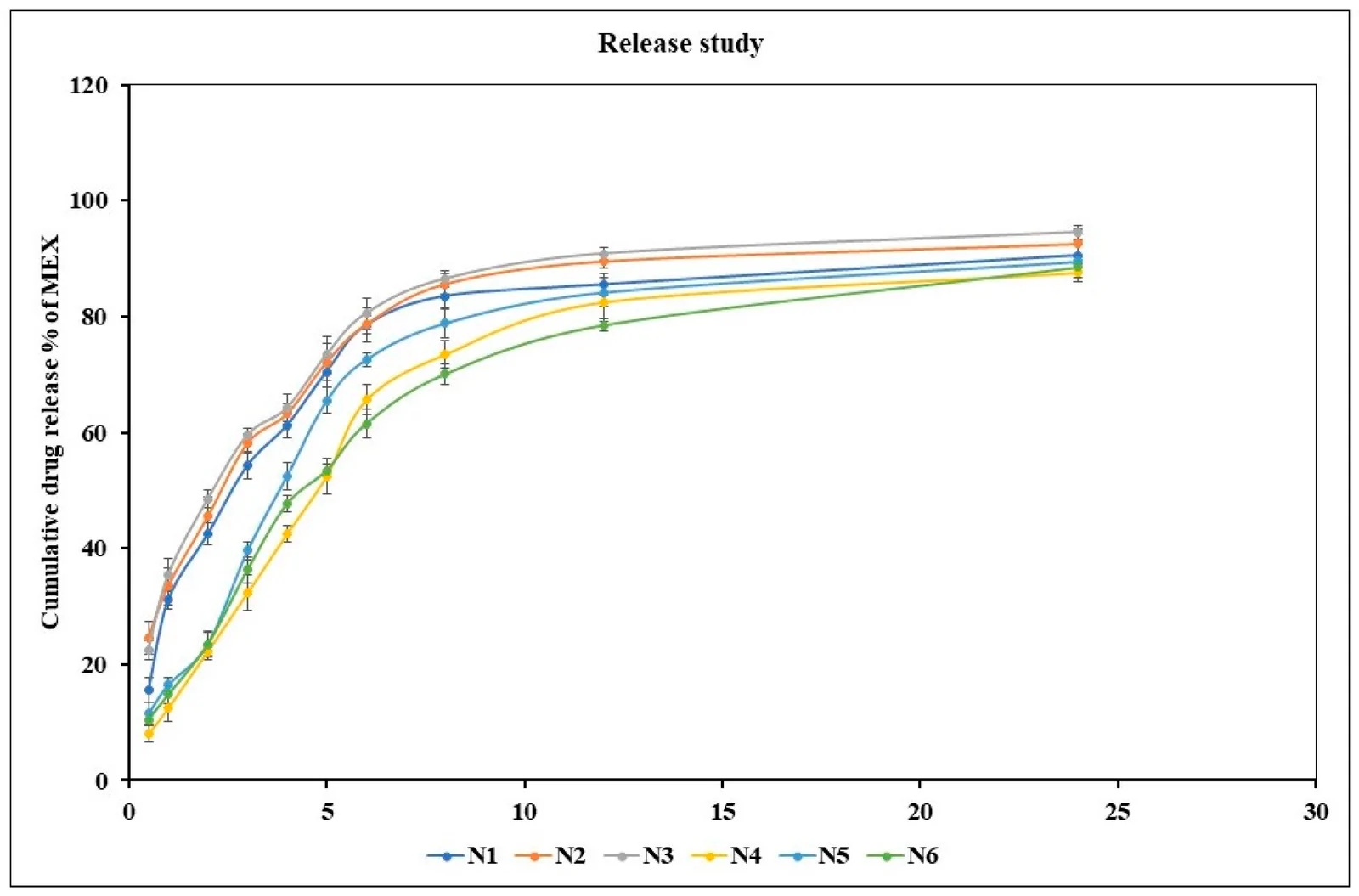
In vitro release profiles of Mexil from the prepared niosomal dispersions in phosphate buffer (pH 6.8) for 24 h.

The in vitro release profiles of niosomal Mexil from N1–N6 exhibited a biphasic pattern characterized by an initial burst release followed by a sustained release phase over 24 h (Figure 4). Cumulative niosomal Mexil release varied among formulations, with N3 showing the highest release (94.5 ± 1.2%), followed by N2 (92.5 ± 1.5%), N1 (90.5 ± 1.7%), N5 (89.5 ± 4.5%), N6 (88.5 ± 3.2%), and N4 (87.6 ± 2.8%). 

The faster release from N3 could be due to its lower EE%, which leads to a larger readily releasable drug fraction. In contrast, N4 exhibited the slowest release, which correlates with its high EE%, relatively rigid and stable vesicular structure, and low PDI. Thus, variations in release behavior among the formulations can be attributed to differences in drug entrapment, bilayer composition, membrane rigidity, and permeability. The sustained release phase is consistent with diffusion of entrapped Mexil through the niosomal bilayer, with possible contribution from vesicular membrane relaxation, as described by the Higuchi and Korsmeyer–Peppas models [28,30,31].

Despite its relatively lower cumulative release, N4 was considered the optimized formulation because it combined high EE%, favorable nanoscale size, high zeta potential, low PDI, and sustained drug release behavior.

### 2.5. Release Kinetics and Mechanism

The in vitro release kinetics showed different mechanisms in the formulations, with N1, N2, N4, and N5 following the Higuchi diffusion-controlled release model, while N3 and N6 followed the first-order kinetics. The fitting of the data to the Korsmeyer–Peppas model yielded release exponents (n) in the range of 0.471 to 0.825 for N1, N2, N4, and N5, indicating anomalous (non-Fickian) transport [32,33]. This pattern suggests a combination of drug diffusion through the lipid bilayer and membrane relaxation or matrix hydration [28,33]. All of the formulations best fitted to the Higuchi model (R2 = 0.875), with N4 having an n-value of 0.825, approaching non-Fickian relaxation-dominated release. High conformity to Higuchi kinetics, along with the near-anomalous transport, confirms that N4 offers sustained matrix diffusion-controlled drug delivery [26,32], which justifies the choice of N4 as the optimized formulation.

### 2.6. Storage Effect

The optimized Mexil-loaded niosomal formulation (N4) exhibited good physicochemical stability during storage for 6 months at refrigerated storage (5 ± 3 °C) and at ambient conditions (25 ± 2 °C). Under refrigerated storage, negligible changes were observed in the mean particle size (205.6–204.6 nm), PDI (0.393–0.385), zeta potential (−60.5–−62.5 mV), and EE% (93.0–92.0%), indicating good vesicular integrity and resistance to drug leakage. However, storage at room temperature resulted in a moderate increase in vesicle size (224 nm) and PDI (0.452) while maintaining strong negative surface charge (−62.5 mV) and high drug retention (91.2%). The minor particle growth observed at room temperature is a known phenomenon in vesicular systems; higher temperatures increase kinetic energy and molecular mobility, resulting in enhanced collision frequency, vesicle aggregation, or Ostwald ripening over time [32,33]. Lower storage temperatures (4–5 °C) were found to suppress the kinetic energy of vesicles and to fluidize the lipid membrane to a smaller extent, hence limiting aggregation or fusion events [28,33]. Yet a high negative surface charge (−62.5 mV) was maintained, providing sufficient electrostatic repulsion to prevent severe instability or macro-aggregation, indicating that N4 has acceptable physicochemical stability under both storage conditions.

### 2.7. The Effect of Niosomal Mexiletine on Rotenone-Induced Motor Impairment

**Figure 5:**
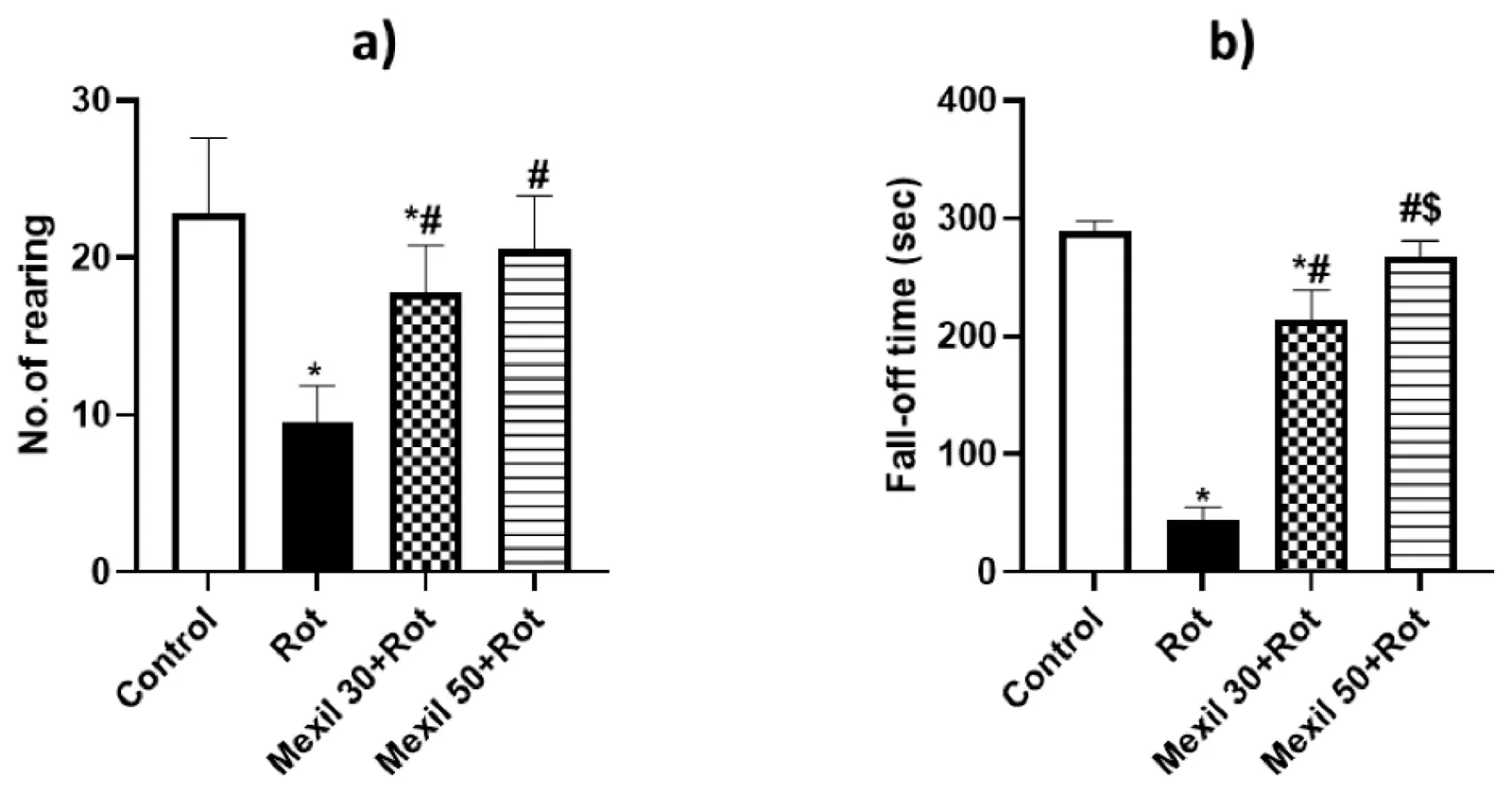
Niosomal mexiletine counteracts rotenone-evoked deficits in spontaneous motor activity and motor coordination. (**a**) Number of rearing; (**b**) Fall-off time. Measures are summarized as mean ± SD (*n* = 12). The statistical differences have been examined using one-way ANOVA with Tukey’s post hoc multiple comparison analysis, *p* < 0.05. * Significant variance vs. control group; # vs. Rot group; $ vs. Mexil 30 + Rot group.

As illustrated in Figure 5, the treatment of Rot significantly reduced motor function in rats, as indicated by a substantial decline in the number of rearing by 58.2% against the control group (*p* < 0.05). Treatment with niosomal Mexil at doses of 30 and 50 mg/kg considerably improved spontaneous motor activity, with an increase in the rearing numbers by 86% and 1.15-fold, respectively, compared to the rotenone-treated rats (*p* < 0.05).

The rotarod test was also exploited to measure muscle incoordination. Rotenone-treated rats exhibited a noteworthy reduction in fall-off time, with an 85% reduction in duration on the rotarod compared with the control group (*p* < 0.05). Treatment with niosomal Mexil at 30 and 50 mg/kg considerably improved this deficit, with 3.8- and 5-fold increases in time on the rotarod, respectively, compared with the Rot group (*p* < 0.05).

### 2.8. The Impact of Niosomal Mexiletine on α-Synuclein, Dopamine, and Tyrosine Hydroxylase

**Figure 6:**
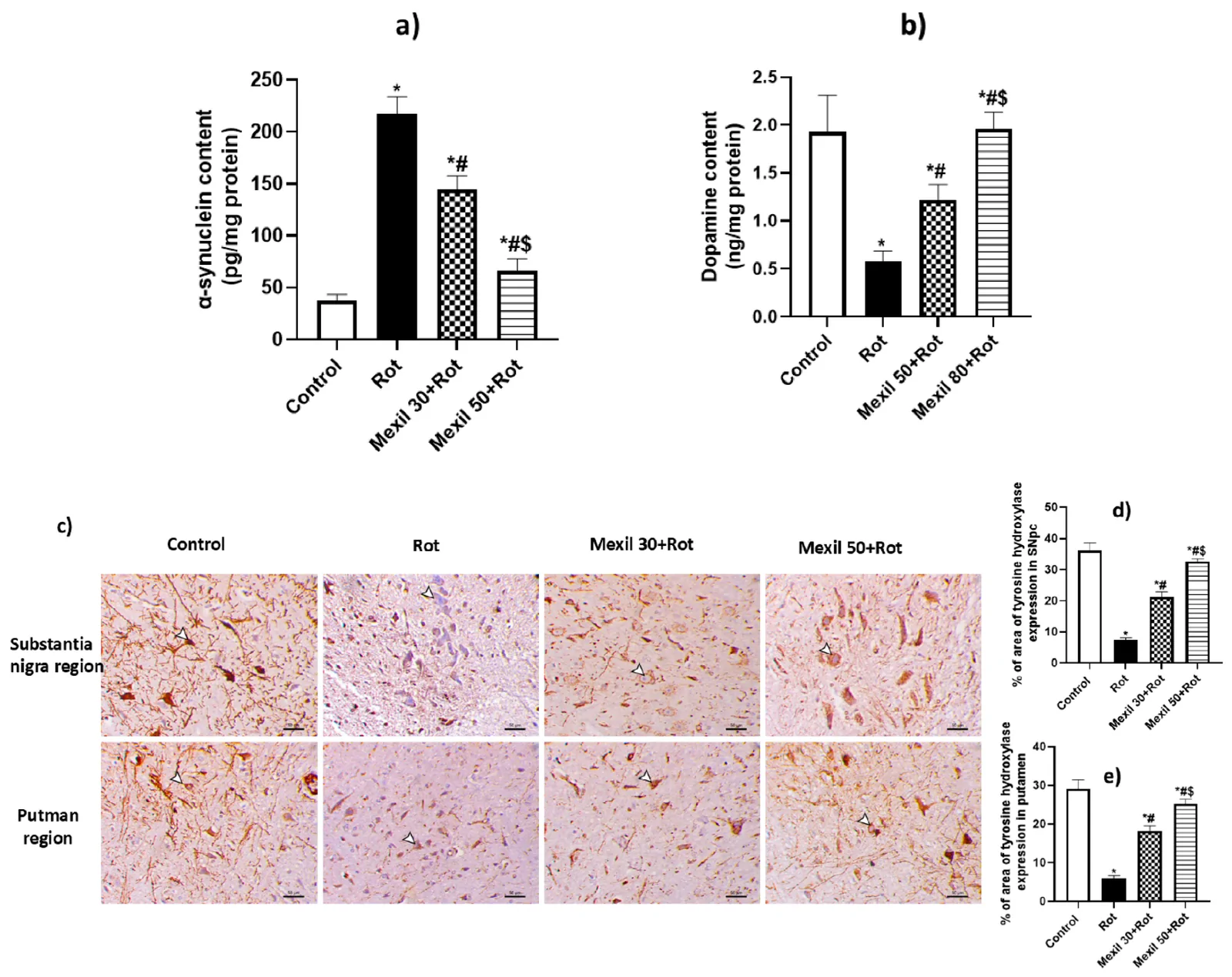
Niosomal mexiletine attenuates α-synuclein accumulation and preserves dopamine and tyrosine hydroxylase (TH) expression in the midbrain of rotenone-treated rats. (**a**) α-synuclein; (**b**) dopamine content; (**c**) representative photomicrography of tyrosine hydroxylase expression in substantia nigra (SN) and putamen regions, scale = 50 µm; (**d**) % of area expression of tyrosine hydroxylase in SN; (**e**) % of area expression of tyrosine hydroxylase in putamen. Measures are summarized as mean ± SD (*n* = 6). White arrow heads refer to expression of TH. The statistical differences have been examined using one-way ANOVA with Tukey’s post hoc multiple comparison analysis, *p* < 0.05. * Significant variance vs. control group; # vs. Rot group; $ vs. Mexil 30 + Rot group.

Rotenone (Rot) exposure was associated with a significant improvement in midbrain content of α-synuclein by 4.8-fold in the control group (*p* < 0.05). Administration of niosomal Mexil (30 and 50 mg/kg) significantly attenuated this alteration by reducing α-synuclein content by 33.4% and 69.5% relative to the Rot group (*p* < 0.05) (Figure 6a).

This was complemented by a significant reduction in midbrain dopamine content, which declined by 70.3% compared to the control group (*p* < 0.05). Contrary to this, treatment with niosomal Mexil at doses of 30 and 50 mg/kg significantly ameliorated this depletion by increasing dopamine content by 1.13-fold and 2.4-fold, respectively, relative to the rotenone-treated group (*p* < 0.05; Figure 6b).

Moreover, Figure 6d,e illustrates significant attenuation in the substantia nigra and putamen regions’ expression of tyrosine hydroxylase by 79% and 79.5%, respectively, relative to the control group, at *p* < 0.05. Nevertheless, treatment with niosomal Mexil (30, 50 mg/kg) illustrated significant enhancement in expression of tyrosine hydroxylase in substantia nigra and putamen by 1.81, 3.3, 2.1, and 3.2-fold, respectively, in the rotenone-treated rats at *p* < 0.05.

### 2.9. The Attenuation Effect of Niosomal Mexiletine on Oxidative Stress Induced by Rotenone Administration

**Figure 7:**
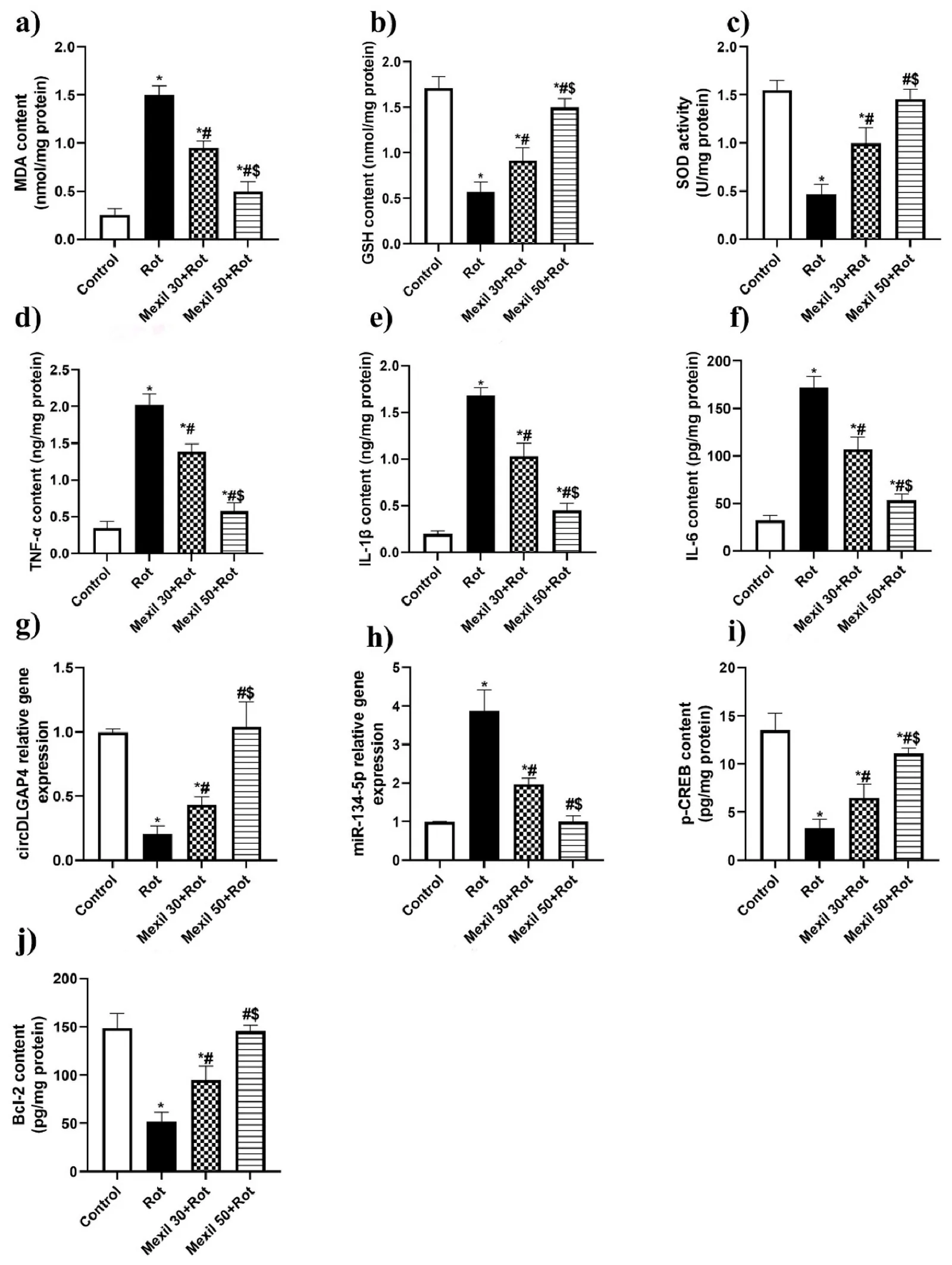
Niosomal mexiletine attenuates oxidative stress induced in the midbrain of rotenone-treated rats. (**a**) MDA; (**b**) GSH content; (**c**) SOD activity; (**d**) TNF-α content; (**e**) IL-1β content; (**f**) IL-6 content; (**g**) relative expression of circDLGAP4; (**h**) relative expression of miR-134-5p; (**i**) p-CREB; (**j**) Bcl-2 content. Measures are summarized as mean ± SD (*n* = 6). The statistical differences have been examined using one-way ANOVA with Tukey’s post hoc multiple comparison analysis, *p* < 0.05. * Significant variance vs. control group; # vs. Rot group; $ vs. Mexil 30 + Rot group.

As depicted in Figure 7a–c, Rot administration induced significant oxidative stress in the midbrain, characterized by a 4.8-fold increase in MDA content and a 66.5% and 69.8% decrease in GSH content and SOD activity, respectively, in comparison with the control group (*p* < 0.05). Niosomal Mexil administration (30 and 50 mg/kg) significantly attenuated these oxidative changes, with a decline in MDA content and restoration of GSH content and SOD activity. Specifically, MDA content was reduced by 57.6% and 66.7%, respectively, while GSH content was increased by 59.2% and 1.6-fold and SOD activity improved by 1.12-fold and 2.11-fold in the Rot-treated group (*p* < 0.05).

### 2.10. Influence of Niosomal Mexil on Inflammatory Response Induced by Rotenone Administration

Rot administration significantly enhanced the inflammatory response, as revealed by significant increases in the midbrain contents of TNF-α, IL-1β, and IL-6 by 4.5-, 7.5-, and 4.4-fold, respectively, compared to the control group (*p* < 0.05) (Figure 7d–f). Treatment with niosomal Mexil (30 and 50 mg/kg) significantly attenuated this inflammatory response. Specifically, TNF-α content was reduced by 31.6% and 73.3%, IL-1β by 71.6% and 38.0%, and IL-6 by 38.6% and 69.0%, respectively, compared to the rotenone-treated rats.

### 2.11. The Impact of Niosomal Mexiletine on Expression of circDLGAP-4, miR-134-5p, and p-CREB in Rotenone-Treated Rats

As shown in Figure 7g–j, rotenone administration significantly downregulated circDLGAP4 expression in the midbrain by 78.5% while markedly upregulating miR-134-5p expression by 2.9-fold in contrast to the control group (*p* < 0.05). These alterations were associated with a significant 74% reduction in p-CREB content and a 66% reduction in Bcl-2 content relative to the control group (*p* < 0.05).

Treatment with niosomal Mexil (30 and 50 mg/kg) substantially reversed these molecular changes. CircDLGAP4 expression increased by 1.1-fold and 3.9-fold, respectively, whereas miR-134-5p expression was reduced by 50.0% and 73.5%, respectively, compared with the Rot group (*p* < 0.05). In parallel, p-CREB levels were significantly elevated by 88% and 2.1-fold, respectively, compared with rotenone-treated rats (*p* < 0.05). Additionally, Bcl-2 content significantly increased in the midbrain levels of the mexil-treated groups (30 and 50 mg/kg) by 88% and 1.9-fold, respectively, compared to the Rot group, at *p* < 0.05.

### 2.12. Effect of Niosomal Mexiletine on Improvement Mitochondrial Function in Rotenone-Treated Rats

**Figure 8:**
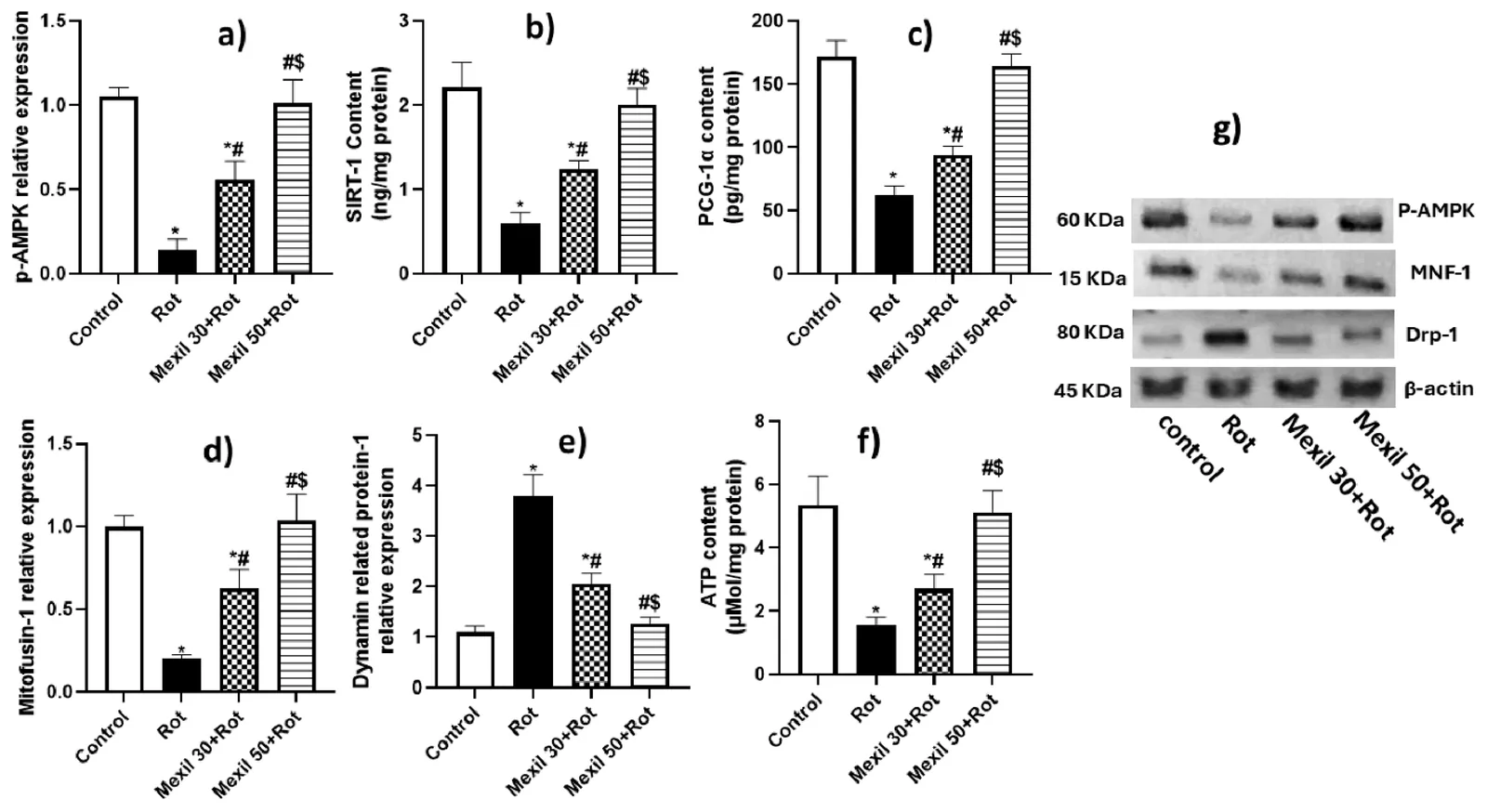
Significance of effect of niosomal mexiletine on expression of mitochondrial function in rotenone-treated rats. (**a**) Relative expression of p-AMPK; (**b**) SIRT-1 level; (**c**) PGC-1α level; (**d**) Mitofusin-1 relative expression (MFN-1); (**e**) dynamin related protein-1 relative expression; (**f**) ATP content; (**g**) representative blot for expression of p-AMPK, MFN-1, and Drp-1. Measures are summarized as mean ± SD (*n* = 6). The statistical differences have been examined using one-way ANOVA with Tukey’s post hoc multiple comparison analysis, *p* < 0.05. * Significant variance vs. control group; # vs. Rot group; $ vs. Mexil 30 + Rot group.

Rot treatment caused significant suppression of the p-AMPK/SIRT-1/PGC-1α signaling pathway, indicating significant damage to mitochondrial function in the Rot group. The expression of p-AMPK in the midbrain and the level of SIRT-1 were significantly reduced by 86.7% and 73%, respectively, and the expression of PGC-1α was significantly downregulated by 63.5% after administration of rotenone compared with the control group (*p* < 0.05). This is reinforced by detecting the expression of mitofusin-1 (MFN-1) and dynamin-related protein-1 (Drp-1) in the Rot group, which showed a significant reduction in the expression of midbrain MFN-1 by 79.8% and augmentation in the expression of Drp-1 in the midbrain by 2.45-fold compared to the control group, at *p* < 0.05. This consequently led to obvious depletion in the midbrain content of ATP by 70.35% in the Rot group compared to the control group (Figure 8).

Treatment with niosomal Mexil (30 and 50 mg/kg) effectively improved rotenone-induced mitochondrial signaling impairment. Both doses significantly increased midbrain p-AMPK expression by 3- and 6.3-fold, respectively, and increased SIRT-1 levels by 1.06- and 2.4-fold, respectively, compared to the Rot group (*p* < 0.05). Consequently, PGC-1α content was significantly increased by 49.5% and 1.62-fold, respectively, compared to the Rot group (*p* < 0.05) (Figure 8a–c). Moreover, niosomal Mexil significantly improved mitochondrial dynamics, as demonstrated by a significant increase in midbrain MFN-1 expression by 2.12- and 4.2-fold, respectively, but Drp-1 expression was significantly reduced by 45.6% and 66.3%, respectively, compared to the Rot group (*p* < 0.05). Furthermore, niosomal Mexil treatment significantly ameliorated mitochondrial bioenergetics, as demonstrated by a significant increase in midbrain ATP content by 71% and 2.25-fold, respectively, compared to the Rot group (*p* < 0.05) (Figure 8d–f).

### 2.13. Effect of Niosomal Mexiletine on Autophagy Induction in Rotenone-Treated Rats

**Figure 9:**
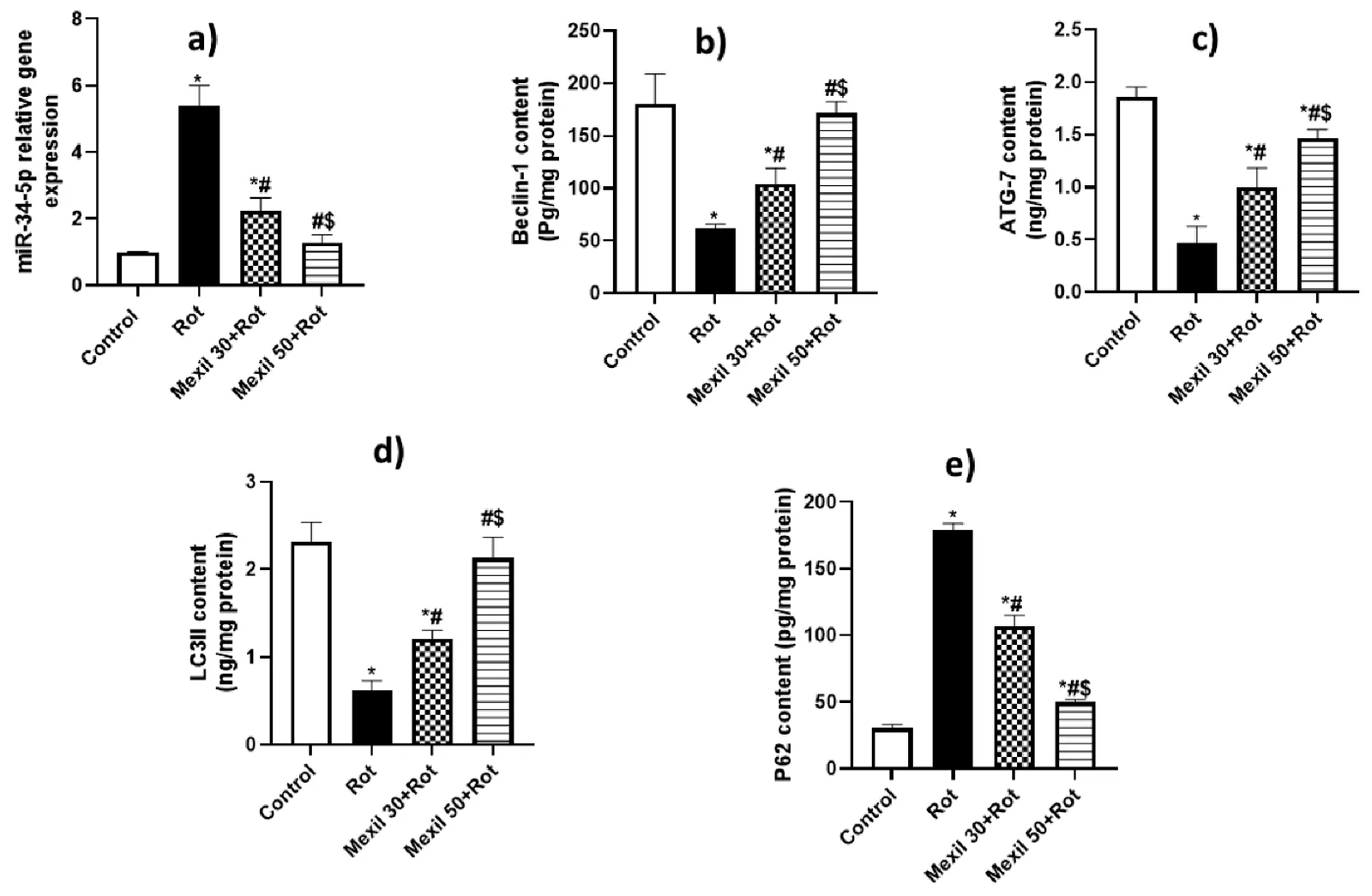
Niosomal mexiletine restored autophagy in rotenone-treated rats. (**a**) Relative expression of miR-34a-5p; (**b**) Beclin-1 level; (**c**) ATG-7 level; (**d**) LC3II level; (**e**) P62 level. Measures are summarized as mean ± SD (*n* = 6). The statistical differences have been examined using one-way ANOVA with Tukey’s post hoc multiple comparison analysis, *p* < 0.05. * Significant variance vs. control group; # vs. Rot group; $ vs. Mexil 30 + Rot group.

The effect of Rot treatment was profound on the miR-34a-5p/Beclin-1/ATG-7/LC3II/p62 signalling pathway, thus indicating a severely impaired autophagic axis in the midbrain. Rot treatment significantly increased miR-34a-5p expression by 4.43-fold and concomitantly decreased Beclin-1 content by 65.8% compared to the control group (*p* < 0.05). Consistent with this impairment, the downstream autophagy markers ATG-7 and LC3II were significantly decreased by 74.5% and 73%, respectively. Additionally, the level of p62 in Rot group was significantly elevated by 4.8-fold compared to the control group, thus pointing out defective autophagosome formation and autophagic activity in the Rot group (*p* < 0.05) in Figure 9.

The impaired autophagy signaling pathway induced by rotenone was significantly restored after treatment with niosomal Mexil (30 and 50 mg/kg). Niosomal Mexil treatment reduced midbrain miR-34a-5p expression significantly by 58.5 and 76.2% and remarkably increased Beclin-1 content by 69.5% and 1.8-fold, respectively, in comparison to the Rot group (*p* < 0.05). The restoration of the miR-34a-5p/Beclin-1 axis was associated with a significant improvement of the downstream autophagy-related proteins, as reflected by increased ATG-7 expression by 1.1- and 2.2-fold and LC3II content by 93.3% and 2.4-fold and a decrease in the content of p62 by 40.6% and 72% respectively, in comparison to the Rot group (*p* < 0.05), which indicate the reactivation of the autophagic machinery following niosomal Mexil treatment.

### 2.14. Niosomal Mexiletine Restored Histopathological Alteration Induced by Rotenone Administration

**Figure 10:**
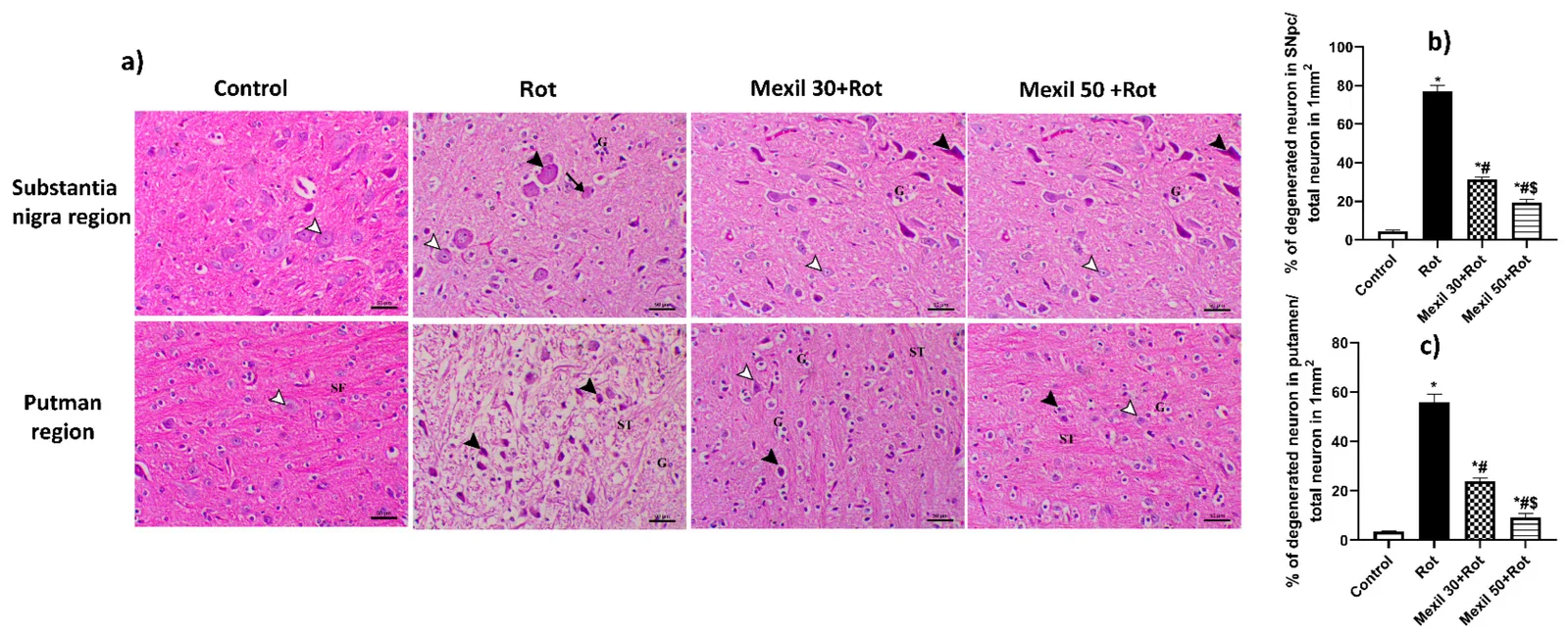
Histopathological assessment of the substantia nigra pars compacta and putamen in rotenone-induced Parkinsonian rats and the protective effect of niosomal mexiletine. Representative photomicrographs (**a**) showing the histopathological alterations in the substantia nigra pars compacta (SNpc) and putamen across the experimental groups. The control group showed normal neuronal architecture, whereas rotenone-treated rats exhibited marked neuronal degeneration, cellular swelling, tigrolysis (black arrowhead), diffuse gliosis with neuronophagia (G), and intracytoplasmic Lewy bodies (arrow) in the SNpc. Niosomal Mexil treatment (30 and 50 mg/kg) progressively attenuated these pathological changes, with increased numbers of normal neurons (white arrowhead) and reduced neuronal degeneration and gliosis (black arrowhead, G). In the putamen, Rot induced severe ischemic and liquefactive neuronal degeneration (black arrowhead), gliosis, and marked necrotic/axonal degeneration of striatal fibers (ST). Meanwhile, niosomal Mexil treatment preserved neuronal integrity and striatal fibers (SF/ST) and increased the number of intact neurons (white arrowhead), with only few glial cells (G) remaining at 50 mg/kg, scale = 50 µm; (**b**) % of degenerated neurons in SPnc region; (**c**) % of degenerated neurons in putamen region. Measures are summarized as mean ± SD (*n* = 6). The statistical differences have been examined using one-way ANOVA with Tukey’s post hoc multiple comparison analysis, *p* < 0.05. * Significant variance vs. control group; # vs. Rot group; $ vs. Mexil 30 + Rot group.

Histopathological examination of the substantia nigra pars compacta (SNpc) revealed a control animal showing normal pyramidal neurons with abundant eosinophilic cytoplasm containing basophilic Nissl granules and centrally located vesicular nuclei. Rotenone-treated animals showed severe degenerative ischemic neuronal injury characterized by marked cellular swelling, pronounced tigrolysis (black arrowhead), and diffuse gliosis associated with neuronophagia (G); intracytoplasmic Lewy bodies are also evident (arrow). Rats treated with niosomal Mexil (30 mg/kg) showed focal ischemic degenerative changes in neuronal cells (black arrowhead) accompanied by mild gliosis (black arrow) and a remarkable increase in the number of morphologically normal neurons (white arrowhead). The diseased animal treated with niosomal Mexil (50 mg/kg) showed a marked reduction in ischemic neuronal degeneration accompanied by decreased gliosis (G) (black arrowhead) and a marked increase in the number of morphologically normal neurons (white arrowhead), Figure 10a.

Histopathological examination of the putamen region showed normal neuronal cells (white arrowheads) and intact striatal fibers (SF) in the control group. In contrast, the diseased group exhibited severe ischemic degenerative and liquefactive changes in neuronal cells (black arrowheads) accompanied by prominent gliosis. This marked necrotic and axonal degenerative changes of the striatal fibers (ST). Niosomal Mexil treatment at a dose of 30 mg/kg minimized these pathological changes, as indicated by a reduced number of ischemic degenerated neurons (black arrowheads), maintenance of the striatal fibers (ST), and an increased number of morphologically normal neurons (white arrowheads). The niosomal Mexil 50 mg/kg treated group showed more pronounced histological improvement characterized by a marked increase in the number of intact neurons (white arrowheads), only a few degenerated neurons (black arrowheads), and maintenance of normal striatal fibers; a few glial cells (G) were also observed (Figure 10a).

All of these are confirmed by scoring degenerated neurons in the SNpc and putamen region, which showed a remarkable increase in degenerated neurons by 16- and 15.1-fold, respectively, compared to control rats (*p* < 0.05) (Figure 10b,c).

However, treatment with 30 and 50 mg/kg of niosomal Mexil caused a substantial decrease in the percentage of degenerated neurons/total neurons in 1 mm^2^ in the SNpc region by 59.5% and 74.8%, respectively. Moreover, an obvious attenuation in the percentage of degenerated neurons / total neurons in 1 mm^2^ was detected in the putamen region by 57.2% and 83.25%, respectively, compared to the Rot group at *p* < 0.05 (Figure 10b,c).

### 2.15. In Silico Molecular Docking Evaluation of the Mexiletine

Molecular docking studies were conducted to investigate the mechanical aspects of the mexil in conjugation with CREBBP and alpha-synuclein using PDB IDs 3P1C and 7WMM. To ensure the robustness of the docking protocol, we first performed a redocking validation using the crystallized ligand (PDB IDs: ALY and 1KI) into the CREBBP and alpha-synuclein active site, respectively. The redocked pose reproduced experimental geometry with an RMSD of 1.25 and 1.26 Å and a binding score of −5.2 and −4.9 kcal · mol^−1^.

Using the validated protocol, mexil and standard molecules were docked into the binding pocket of two proteins. Visual inspection of the superposed poses shows that they occupy the canonical binding cavity and substantially overlap the crystallized ligands (PDB IDs: ALY and 1KI) into the binding cavity, indicating consistent targeting of crucial residues for CREBBP and alpha-synuclein binding pockets, The validation and docking study results are summarized in Table 1.

## 3. Discussion

Parkinson’s disease (PD) is a common neurodegenerative disease with an estimated prevalence of approximately 1–2% in those over 60 years of age [34]. PD is characterized by progressive motor deficits, including resting tremors, rigidity, bradykinesia, and impaired coordination. In the present study, the rotenone-induced motor deficits were comprehensively assessed using the cylinder and rotarod tests. Because impaired coordination is a key feature of PD and a clinically relevant measure of therapeutic efficacy, these complementary behavioral assessments provided a robust evaluation of motor function. Collectively, the current results revealed that niosomal Mexil significantly improved motor coordination and reduced behavioral deficits in rotenone-induced rats, suggesting its possible therapeutic utility in PD.

The behavioral recovery elicited by niosomal Mexil was paralleled by a broader restoration of neuronal homeostasis. Treatment attenuated oxidative and inflammatory responses while regulating the circDLGAP4/miR-134-5p/CREB axis. Niosomal Mexil also rebalanced mitochondrial dynamics, as reflected by reduced Drp1 expression and increased mitofusin levels, alongside activation of the p-AMPK/SIRT1/PGC-1α pathway. These changes coincided with enhanced autophagic clearance and reduced apoptotic signalling. Taken together, the findings suggest that niosomal Mexil mitigates rotenone-induced neurodegeneration through coordinated regulation of redox balance, inflammation, mitochondrial integrity, autophagy, and neuronal survival.

This neuroprotective effect may be partly attributed to the antioxidant property of mexiletine, as previously reported, and may also be facilitated by the niosomal delivery system. Niosomal formulations have become promising nanocarrier-based approaches to improve the delivery of therapeutic agents to the central nervous system. Niosomes are mainly composed of non-ionic surfactants and cholesterol that may have beneficial effects on drug stability, controlled release, and tissue distribution, which in turn may increase therapeutic exposure in the brain. They have been investigated as delivery vehicles for a broad range of therapeutic cargos, including conventional drugs, peptides, antisense oligonucleotides, and RNA-based therapeutics, with potential applications in neurodegenerative disorders such as Parkinson’s and Alzheimer’s diseases [35,36].

Daily subcutaneous administration of Rot (2 mg/kg) for five weeks established a reproducible Parkinsonian phenotype. Motor impairment was accompanied by α-synuclein accumulation, loss of tyrosine hydroxylase-positive neurons in the substantia nigra pars compacta, and a marked reduction in striatal dopamine, consistent with the findings of Zhang et al. [1]. This neurochemical profile provides a clear explanation for the behavioral deficits, given the central role of striatal dopamine in motor control [37]. Moreover, the reduction in tyrosine hydroxylase—the rate-limiting enzyme in dopamine biosynthesis—further indicates disruption of nigrostriatal dopaminergic integrity. Similar associations between motor dysfunction, dopamine depletion, and reduced tyrosine hydroxylase have been reported in experimental PD models [2,38].

Circular RNAs can regulate gene expression by sequestering microRNAs and limiting their interaction with downstream targets [39]. In this context, circDLGAP4 has been identified as a molecular sponge for miR-134-5p, implicating the circDLGAP4/miR-134-5p axis in PD-related cellular processes. Previous evidence supported a neurotoxic role for miR-134-5p, including the promotion of oxidative and endoplasmic reticulum stress, apoptosis, and aberrant mossy-fiber sprouting in epilepsy [40], as well as neuronal death following cerebral ischemia–reperfusion injury [41]. Conversely, suppression of miR-134-5p has been associated with improved learning and memory in aged mice treated with resveratrol [42]. Although its involvement in PD was previously unclear, miR-134-5p was found to be upregulated in 1-methyl-4-phenyl-1,2,3,6-tetrahydropyridine (MPTP)-treated mice and MPP^+^-exposed cellular models, where it contributed to neuronal injury. These observations support the circDLGAP4/miR-134-5p axis as a relevant regulator of PD-associated neurodegeneration [6].

Mechanistically, CREB was validated as a downstream target of miR-134-5p, and circDLGAP4 sustained CREB expression by sponging miR-134-5p, thereby avoiding its repressive effect. CREB is a cAMP responsive transcription factor, and its activity is potentiated by phosphorylation at Ser133 [43]. Upon activation, CREB enhances neural resilience via transcriptional control of many survival-associated genes, including BDNF, Bcl-2, and PGC-1α [43,44,45]. BDNF promotes neuronal development, survival, synaptic plasticity, and neurotransmission [46]. Bcl-2 inhibits mitochondrial apoptosis by blocking cytochrome-c release, followed by caspase activation [6]. PGC-1α also regulates mitochondrial biogenesis, antioxidant defence and the production of mitochondrial uncoupling proteins, therefore contributing to neural homeostasis [47]. Thus, control of the circDLGAP4/miR-134-5p/CREB axis might affect neuronal lifespan through integration of neurotrophic support, mitochondrial maintenance, antioxidant capability, and apoptosis resistance.

In agreement with this regulation model, Rot significantly dysregulates the circDLGAP4/miR-134-5p/CREB axis, along with corresponding declines in Bcl-2 and PGC-1α expression. Treatment with niosomal Mexil modulates these changes, which suggests that the restoration of CREB-dependent signaling contributes to its mitochondrial, anti-apoptotic, and neuroprotective benefits. Together, these results suggest that the activation of the circDLGAP4/miR-134-5p/CREB axis is involved in niosomal Mexil-induced neuroprotection against rotenone administration.

The AMPK/SIRT1/PGC-1α axis is one of the signaling networks involved in mitochondrial homeostasis and plays a critical role in linking cellular energy status to metabolic adaptability. The activation of AMPK is mediated by increasing ratios of AMP/ATP, whereas SIRT1 is sensitive to NAD^+^ levels and redox status [48]. These energy sensors converge on PGC-1α, a transcriptional coactivator that regulates mitochondrial biogenesis, oxidative phosphorylation and antioxidant defense through phosphorylation- and deacetylation-dependent pathways [48,49]. Dysfunction of this axis is directly related to the etiology of Parkinson’s disease. Reduced AMPK phosphorylation in the substantia nigra impairs neural responses to metabolic stress. AMPK activation promotes clearance of α-synuclein by increasing ULK1-dependent autophagy, blocking mTORC1, and activating TFEB-mediated lysosomal biogenesis [50,51,52,53]. Simultaneous inhibition of SIRT1 worsens proteostatic collapse, mitochondrial dysfunction, oxidative damage and apoptosis [49]. Similarly, lower PGC-1α activity limits mitochondrial biogenesis and ATP production, while its activation enhances the activity of the respiratory chain through NRF1/TFAM signaling. Therefore, the AMPK/SIRT1/PGC-1α axis is a key molecular interface between energy sensing, proteostasis, and mitochondrial integrity in dopaminergic neurons [49].

Consistent with this mechanistic framework, rotenone exposure suppressed the AMPK/SIRT1/PGC-1α axis, indicating impaired mitochondrial biogenesis and metabolic adaptation in the midbrain. This disruption was accompanied by a marked reduction in ATP content, reflecting compromised mitochondrial bioenergetic capacity. These changes were markedly ameliorated by niosomal Mexil with restoration of AMPK/SIRT1/PGC-1α signaling and ATP levels. Our findings provide evidence for the involvement of the AMPK/SIRT1/PGC-1α pathway in maintaining mitochondrial biogenesis and energy homeostasis in niosomal Mexil-mediated neuroprotection against rotenone-induced neurodegeneration.

Mitochondrial dynamics and biogenesis are complementary quality control processes that regulate one another through coordinated crosstalk. Their balanced activity enables cells to adapt to changes in metabolic demands while preserving mitochondrial integrity and cellular homeostasis [10]. The balance of fission and fusion rates dictates the morphology and distribution of mitochondria, which is crucial for cellular homeostasis and responses to stimuli [54]. Disrupted regulation of mitochondrial dynamics favoring fission has been linked to neuronal mortality in age-related neurodegenerative disorders, like PD [55].

Under physiological conditions, Drp1-mediated fission facilitates mitochondrial turnover and quality control, whereas MFN1/2-mediated fusion preserves network integrity and functional connectivity [56,57]. Cellular stressors, including oxidative stress, energy deprivation, and heavy-metal exposure, can disturb this balance and promote excessive mitochondrial fission. Sustained mitochondrial fragmentation subsequently compromises bioenergetic function, favors the accumulation of dysfunctional mitochondria, enhances reactive oxygen species production, and ultimately activates cell-death pathways [58]. Conversely, selective inhibition of aberrant fission may support mitochondrial biogenesis by increasing PGC-1α expression through CaMKII/CREB signaling and enhancing its activity via the AMPK/SIRT1 pathway. The convergence of these signaling axes therefore establishes a coordinated feedback network that functionally links mitochondrial dynamics with mitochondrial biogenesis [10].

Consistent with these pathways, our findings demonstrated that rotenone increased Drp1 expression while reducing mitofusin-1 (MFN-1) expression; notably, niosomal Mexil reversed these alterations, suggesting restoration of mitochondrial dynamics.

circDLGAP4 has been reported to promote autophagy through regulation of the circDLGAP4/miR-134-5p/PTEN signaling axis [59]. Furthermore, miR-34a-5p, a well-characterized p53-responsive tumor suppressor belonging to the miR-34 family, contributes to the regulation of mitochondrial oxidative phosphorylation and ATP generation. Accumulating evidence indicates that persistent miR-34a-5p elevation is involved in several age-related disorders characterized by mitochondrial dysfunction and impaired autophagy. Conversely, inhibition of miR-34a-5p may help restore mitochondrial function and autophagic activity [60].

In the present study, rotenone exposure elicited a pronounced upregulation of miR-34-5p, paralleled by a concomitant suppression of Beclin-1, LC3-II, ATG7 and increase in the content of p62—a pattern consistent with impaired autophagy. Remarkably, niosomal Mexil administration largely restored these markers toward baseline, suggesting that its protective actions may be mediated, at least in part, through modulation of the miR-34a-5p/autophagy axis.

Autophagosome biogenesis relies on two coordinated ubiquitin-like conjugation systems. First, ATG7 activates ATG12, which is transferred to ATG10 and conjugated to ATG5, forming the ATG12–ATG5 complex required for phagophore nucleation. Second, ATG7 similarly activates LC3-I, which is then lipidated via conjugation to phosphatidylethanolamine to generate LC3-II; this lipidated form integrates into the expanding autophagosomal membrane, driving its elongation and closure [15]. Beclin-1 adds a further regulatory layer by forming a complex with class III PI3-kinase Vps34, coordinating autophagosome nucleation and maturation [61]. p62/SQSTM1 is a protein commonly found in the protein inclusions of neurodegenerative disorders and is involved in intracellular protein trafficking and degradation. It acts as a cargo adaptor that recognizes polyubiquitinated proteins and targets them for destruction via the autophagy–lysosomal and ubiquitin–proteasome systems. Since p62 itself is also a substrate of autophagy, impairment of protein degradation may result in the buildup of p62 together with its associated protein cargo, thus contributing to the formation of intracellular protein aggregates [62].

These autophagic changes are closely tied to mitochondrial health. Mitochondria are, somewhat paradoxically, both the chief source of reactive oxygen species and the system that keeps them in check, a dual role that leaves them chronically vulnerable to oxidative damage. This oxidative burden rarely acts alone; it appears interwoven with metabolic impairment, mtDNA damage, fission–fusion imbalance, and defective mitophagy, each reinforcing the others. This interconnectedness has made oxidative stress an attractive therapeutic target in mitochondrial disease [63,64].

Mitochondrial dysfunction and chronic neuroinflammation reinforce each other, each able to trigger and sustain the other. Mitochondrial dysfunction has been repeatedly observed in major neurodegenerative disorders such as Alzheimer’s disease, Parkinson’s disease, and amyotrophic lateral sclerosis. This cycle is driven largely by inflammation itself: chronic inflammatory activity leads to sustained cytokine release, which promotes redox stress and eventually damages mitochondrial DNA. Damaged mitochondria then further reinforce the problem by triggering persistent inflammatory responses that keep the cycle going [65].

Our data confirms this pattern: administration of rotenone led to a significant increase of MDA and a significant decrease of GSH and SOD, consistent with severe oxidative stress, and a concomitant increase of TNF-α, IL-1β and IL-6, consistent with a robust inflammatory response. Niosomal Mexil treatment significantly attenuated this profile, restoring affected markers to baseline levels. Our study compliance with previous literature that stated Rot administration in rats caused upregulation of inflammatory mediators and oxidative stress markers in Parkinson induced model [66,67].

Hematoxylin and eosin staining provided further corroboration of these biochemical findings at the histological level, indicating a significant decrease in the percentage of deteriorated neurons in the niosomal Mexil-treated groups, consistent with a neuroprotective effect. This was accompanied by up-regulation of Bcl-2, the anti-apoptotic marker outlined above, further supporting reduction of neuronal loss. These findings are in line with those of Sirwi et al. [68], who also observed that rotenone injection increases apoptotic markers and decreases the number of intact viable neurons.

Molecular docking studies were conducted to correlate the anti-Parkinsonism activity of mexil with potential targets, including CREBBP and alpha-synuclein, using (PDB IDs 3P1C and 7WMM).

CREBBP, also known as CREB-binding protein (CBP/KAT3A), is a transcriptional coactivator and histone acetyltransferase that interacts with phosphorylated CREB to facilitate activity-dependent transcription of genes involved in neuronal survival, synaptic plasticity, neurotrophic signaling, and cellular adaptation to oxidative and metabolic stress [69]. These functions are particularly relevant to Parkinsonism, in which dopaminergic neurons are exposed to sustained oxidative stress, mitochondrial dysfunction, impaired neurotrophic support, neuroinflammation, and progressive synaptic and neuronal loss. Impaired CREB–CREBBP signaling may therefore reduce the transcription of protective genes, including those involved in neuronal maintenance and stress resistance, thereby increasing dopaminergic neuronal vulnerability. Accordingly, CREBBP was selected as an exploratory target related to the CREB signaling changes observed in the present rotenone-induced model [70]. The CREBBP bromodomain structure in complex with an acetylated-lysine ligand (PDB ID: 3P1C; 1.82 Å resolution) was chosen because it provides a high-resolution, experimentally defined acetyl-lysine recognition pocket suitable for structure-based docking and re-docking validation [71]. Importantly, CREBBP is a transcriptional coactivator distinct from CREB and phosphorylated CREB; therefore, docking to PDB 3P1C was intended to generate a structural hypothesis concerning possible CREBBP modulation in Parkinsonism rather than to demonstrate direct interaction with p-CREB or establish CREBBP as a validated mediator of mexil-induced neuroprotection.

**Figure 11:**
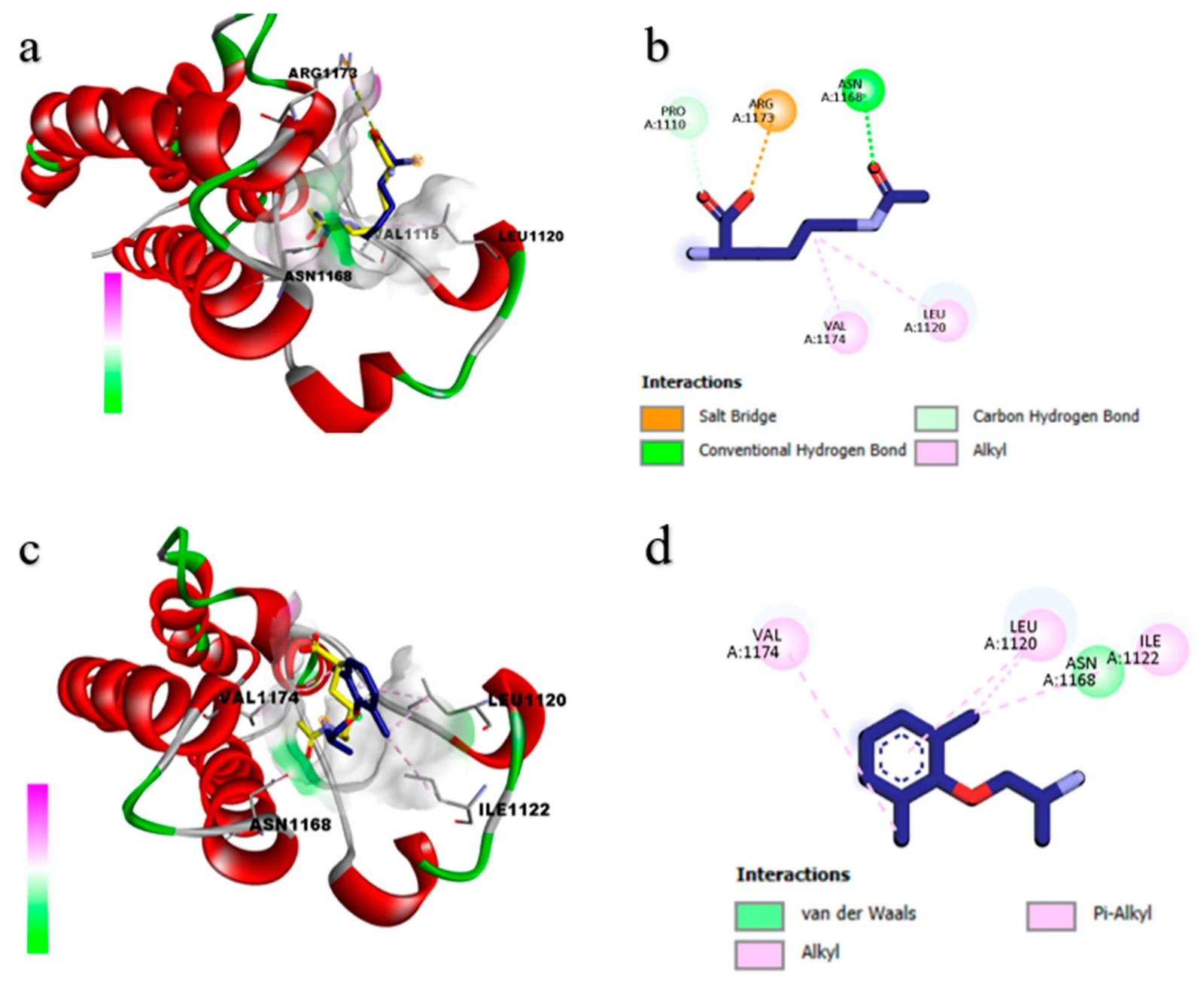
Molecular docking validation and predicted binding mode of mexiletine in CREBBP (PDB ID: 3P1C). (**a**) 3D superposition of the crystallographic ALY ligand (yellow) and its redocked pose (blue). (**b**) 2D interaction map of the crystallographic ALY ligand with CREBBP. (**c**) 3D comparison of the predicted mexiletine pose (blue) and the crystallographic ALY ligand (yellow) within the ALY binding region. (**d**) 2D interaction map of the predicted mexiletine pose with CREBBP. In panels (**a**,**c**), the protein ribbon displays α-helices in red, β-strands in grey, and loops in green. In panel (**b**), dashed lines indicate salt bridges (orange), conventional hydrogen bonds (bright green), carbon–hydrogen bonds (pale green), and alkyl interactions (pink). In panel (**d**), pink dashed lines indicate alkyl and π–alkyl interactions, while pale-green residue shading indicates van der Waals contacts.

Within the CREBBP bromodomain binding pocket, the experimentally resolved acetylated-lysine ligand ALY was stabilized by a salt-bridge interaction with Arg1173, a conventional hydrogen bond with Asn1168, and alkyl contacts with Val1174 and Leu1120. In comparison, mexil occupied the same ligand-associated pocket but exhibited a more limited interaction profile, involving π–alkyl interactions with Val1174 and Leu1120 and van der Waals contacts with Asn1168 and Ile1122. Thus, both ligands were accommodated within a pocket formed primarily by the Arg1173-, Asn1168-, Val1174-, Leu1120-, and Ile1122-containing region (Figure 11).

α-Synuclein is a presynaptic neuronal protein that plays a central role in Parkinson’s disease pathogenesis through misfolding, oligomerization, and fibril formation, processes associated with synaptic dysfunction, mitochondrial impairment, oxidative stress, defective autophagy, neuroinflammation, and dopaminergic neuronal loss [72]. Its accumulation in the rotenone-induced model used in the present study, together with the observed motor deficits, dopamine depletion, tyrosine hydroxylase loss, oxidative imbalance, mitochondrial dysfunction, and impaired autophagic signaling, provides a strong biological rationale for selecting α-synuclein as a disease-relevant molecular target [73]. Accordingly, the experimentally resolved α-synuclein fibril structure containing ligand 1KI (PDB ID: 7WMM) was selected for exploratory docking because it represents a pathological fibrillar state and provides a structurally defined ligand-associated binding groove. This approach was intended to investigate whether mexil could theoretically be accommodated within an α-synuclein-associated pocket; however, the docking findings remain hypothesis-generating and do not establish direct binding or inhibition of α-synuclein aggregation without experimental validation.

**Figure 12:**
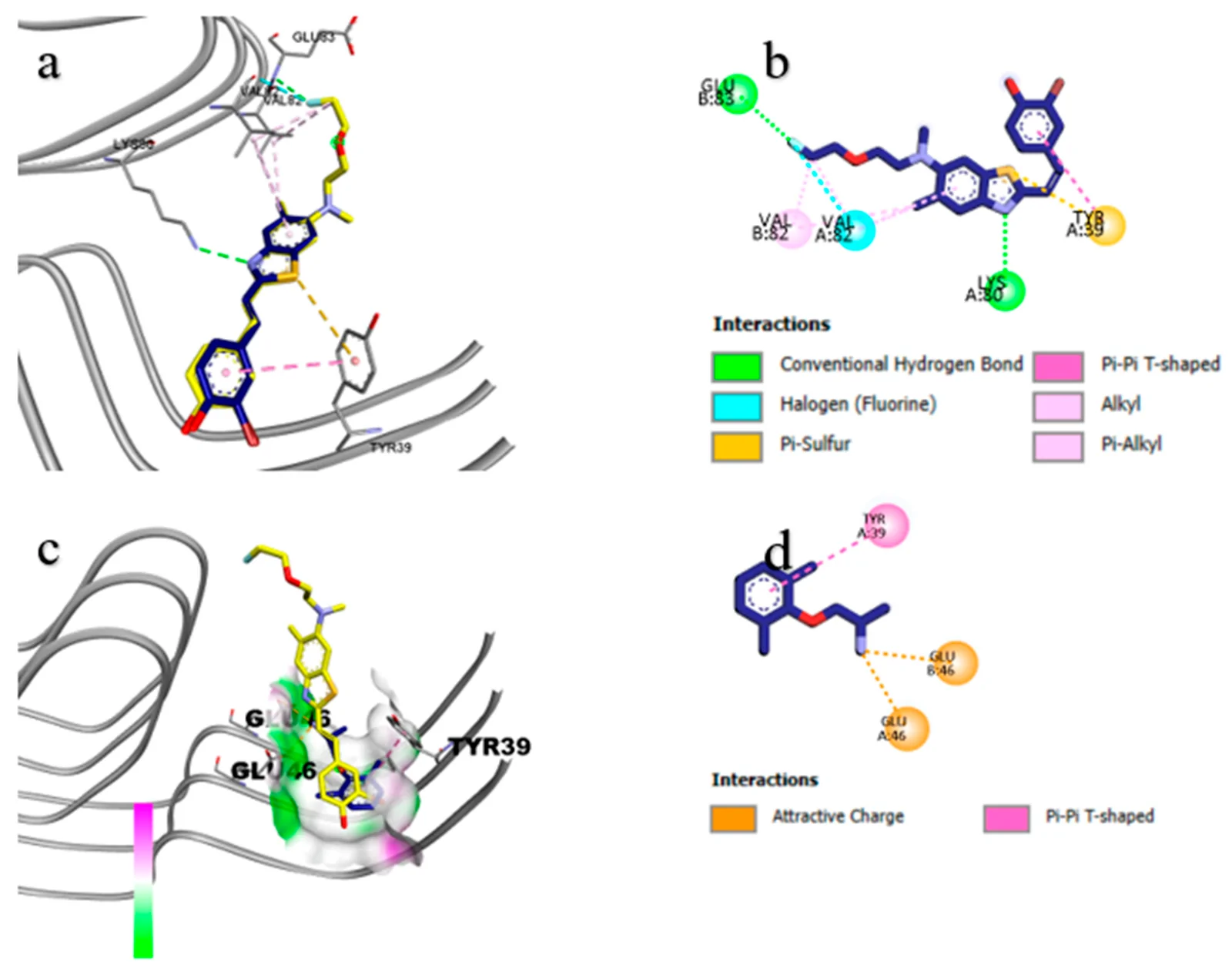
Molecular docking validation and predicted binding mode of mexiletine in the α-synuclein fibril (PDB ID: 7WMM). (**a**) 3d superposition of the experimentally resolved ligand PDB 1KI (yellow color) and its re-docked pose (blue color) in the α-synuclein fibril structure. (**b**) 2d interaction map of the crystallographically resolved ligand 1KI with the α-synuclein binding region. (**c**) 3d representation of the predicted mexiletine pose (blue color) and the experimentally resolved ligand PD:1KI (red color) within the ligand-associated region of α-synuclein. (**d**) 2D representation of the predicted mexiletine interactions with α-synuclein.

The experimentally resolved ligand 1KI occupied the α-synuclein fibril binding pocket through a network of conventional hydrogen-bond, halogen, π–sulfur, π–T-shaped, alkyl, and π–alkyl interactions involving the surrounding residues, including Glu83, Lys80, Val82, and Tyr39. In contrast, mexiletine was predicted to occupy the same ligand-associated groove and to interact primarily through an attractive electrostatic interaction between its protonated amine and Glu46, together with a π–T-shaped interaction involving its aromatic ring and Tyr39. Thus, although both ligands occupy a comparable pocket formed by the Glu83/Val82- and Tyr39-containing region (Figure 12).

The docking results provide a theoretical assessment of the possible accommodation of Mexil within the selected α-synuclein and CREBBP structural models. No direct binding assay was performed in the present study; therefore, the predicted interactions should not be interpreted as experimental evidence of target engagement or as proof of causal involvement in the neuroprotective effects of niosomal Mexil. These findings require validation using orthogonal biochemical and biophysical approaches.

## 4. Material and Methods

### 4.1. FTIR Compatibility and Preparation of Niosomes Loaded with Mexil

The compatibility of Mexil with Span 60, cholesterol (CHL), and lecithin (LEC) was evaluated through FTIR spectroscopy (PerkinElmer Spectrum Two FTIR spectrometer with UATR, 400–4000 cm^−1^, Waltham, MA, USA) [74]. Mexil-loaded niosomes (N1-N6) were developed through the thin film hydration method [75,76] using Span 60, lecithin, and cholesterol. Briefly, excipients were reconstituted in chloroform (2:1, *v*/*v*) and evaporated at 60 °C and 120 rpm for 30 min, and the resulting film was hydrated with Mexil-containing water at 58–60 °C for 1 h (Table 2). The dispersions were probe-sonicated for 3 min and stored at 4 °C. Blank niosomes were prepared similarly without Mexil.

### 4.2. Characterization of Mexil-Loaded Niosomal Dispersions 

#### 4.2.1. Visual Inspection

All formulations were visually examined for color, appearance, homogeneity, and macroscopic aggregation.

#### 4.2.2. Entrapment Efficiency (EE%)

It was determined in triplicate through centrifugation at 20,000 rpm and 4 °C for 1 h, followed by pellet washing, disruption with chloroform, and ethanol dilution. Mexil content was quantified spectrophotometrically at 254 nm against the corresponding blank [77].

$$\mathrm{EE}\%=\frac{\left(Amount\;of\;entrapped\;Mexil\right)}{\left(Original\;amount\;of\;Mexil\;added\right)}\times 100$$

#### 4.2.3. Particle Size, Polydispersity Index (PDI), and Zeta Potential

The colloidal characteristics of the Mexil-loaded niosomal dispersions were measured as mean hydrodynamic diameter, polydispersity index (PDI), and zeta potential using Zetasizer Nano ZS (Malvern Instruments, Malvern Instruments Ltd., Malvern, Worcestershire, UK) at 25 °C. Each formulation was diluted 100-fold with deionized water (1:100, *v*/*v*) for analysis to obtain an appropriate scattering intensity and to reduce the artifacts of multiple scattering in the measurements. Each formulation was measured in three replicates, and the results are represented as mean ± standard deviation (SD; *n* = 3) [78].

#### 4.2.4. Transmission Electron Microscopy (TEM)

The morphological characteristics of the prepared niosomes were examined using a transmission electron microscope (JEOL JEM-2100, JEOL Ltd., Tokyo, Japan). Briefly, an appropriately diluted niosomal dispersion was placed onto a carbon-coated copper grid and allowed to adsorb for approximately 1–2 min. Excess sample was gently blotted with filter paper, and the grid was negatively stained with a suitable staining solution (e.g., 2% PTA in water) for about 1 min. The grid was then air-dried at room temperature prior to examination. TEM visualized samples to evaluate the morphology, vesicular structure, and approximate size of the niosomes. Representative micrographs were taken at suitable magnifications.

#### 4.2.5. In Vitro Drug Release Study

Mexil release was assessed through dialysis using a cellulose membrane (MWCO 12–14 kDa) in phosphate buffer (0.1 M, pH 6.8) at 37 ± 0.5 °C and 100 rpm for 24 h [79,80,81]. At predefined intervals, aliquots were collected, replenished with equal volumes of fresh medium, and assayed at 262 nm. Cumulative Mexil release (%) was calculated from triplicate measurements and plotted against time.

#### 4.2.6. Kinetics and Mechanism of Release

The cumulative release profiles were fitted to zero-order, first-order, Higuchi, and Korsmeyer–Peppas models to characterize the kinetics of Mexil release. The model with the highest coefficient of determination (R 2) was the most appropriate to describe the experimental release behavior [32]. Moreover, the release exponent (n) obtained from the Korsmeyer–Peppas equation was interpreted to provide an insight into the predominant mechanism governing drug liberation from the niosomal system [33].

#### 4.2.7. Stability Study

Niosomal formulations were stored at 5 ± 3 °C and at room temperature for 6 months. Samples were evaluated at 1, 3, and 6 months for PZ, ZP, PDI, and the percentage of entrapped Mexil in the niosomal formulation.

### 4.3. Drugs and Reagents

The purchases of rotenone (Rot) and mexiletine (Mexil) were made at Sigma-Aldrich in St. Louis, MO, USA. Analytical-grade chemicals and reagents comprised the remaining inventory.

### 4.4. Animals

In a controlled environment with a constant temperature of 23 ± 2 °C, a 12 h light/dark cycle, and a humidity of 50 ± 10%, adult male Sprague–Dawley rats weighing an average of 200–250 g were maintained (4 rats/cage). Their diet was supplemented with an unlimited supply of potable water and a conventional pellet diet. Animals were included in the study only if they exhibited normal behavior and were free from any apparent signs of disease or injury. Capital University’s Institutional Animal Care and Use Committee (IACUC) (Protocol number: IACUC 29A2024) authorized this study in accordance with the U.K. Animals (Scientific Procedures) Act, 1986, and related guidelines, which adhere to ARRIVE guidelines. Animals that met pre-determined exclusion criteria defined a priori to the experiment in compliance with the ARRIVE guidelines were excluded from the study. These included poor general health before induction.

#### 4.4.1. Design of an Experimental Study

Randomly, forty-eight male Sprague–Dawley rats were divided into four groups (n = 12) as follows: group I served as the control group, in which the rats received 1 mL/kg/day of sunflower oil s.c. for 35 days, group II served as the rotenone group in which rats received 2 mg/kg/day of rotenone in sunflower oil, s.c. for 35 days [82], group III served as the low dose of niosomal Mexil group in which rats received rotenone (as group II) and niosomal Mexil at a dose of 30 mg/kg, p.o. [83], from day 15 to day 35, and group IV served as a high dose of niosomal Mexil group in which rats received rotenone (as group II) and niosomal Mexil at a dose of 50 mg/kg, p.o. [20], from day 15 to day 35. Sample size determination was dependent on a crude method and previous studies, as no preliminary data or prior pilot studies were available for the present experimental model. Behavioral assessment involving measures for locomotor function and motor coordination was performed on the day after the completion of the treatments.

The randomization process was accomplished through successive allocation of individual rats from the keeper cage and assignment to one of the four treatment groups by lottery (i.e., each rat was placed into a randomly selected cage without replacement until all groups had n = 12).

No animals fulfilled the pre-defined exclusion or humane endpoint criteria during the investigation; therefore, no animals were excluded from the final analysis. No anticipated or unanticipated adverse effects were noted. Humane endpoints were pre-defined and included extreme lethargy, inability to access food or drink, chronic recumbency, or body weight loss of more than 20% of baseline. None of the animals met any of these requirements during the study period.

To minimized potential confounding and bias, all animals were housed under standardized experimental settings with the same handling, treatment administration, and timing of outcome measures for experimental groups. Because of the nature of the intervention, treatment administration could not be conducted under blind settings; however, blinding was maintained during outcome assessment and data analysis. Investigators performing behavioral and biochemical analysis and histological examination were blinded to group allocation. All samples were coded prior to processing. These steps were taken to promote objective outcome evaluation and to reduce the potential for observer-related bias.

#### 4.4.2. Behavior Screening

Behavioral testing was performed in a quiet, dimly lit room between the hours of 1:00 p.m. and 5:00 p.m. Prior to testing, the rats were acclimated to the testing chamber for 60 min. The testing apparatus was cleaned with 70% ethanol between trials to avoid odor signals. Behavioral tests were administered on day 36 of the study regimen, 24 h after the last administration of the treatments, by an investigator blinded to the treatment groups.

#### 4.4.3. Test of the Cylinder

The cylinder test evaluated spontaneous rearing behavior and forelimb motor activity. Individual rats were positioned in a clear Plexiglas cylinder (20 cm diameter, 30 cm height) for 5 min. The number of interactions of the forelimb with the wall of the cylinder was noted as vertical exploration. This treatment was carried out in dim lighting to promote exploratory behavior. A rearing was scored when the forelimbs were raised above shoulder level, contacting the wall of the cylinder with one or both forelimbs. The total number of raising events was calculated for each animal as previously described by Cannon et al. [84].

#### 4.4.4. Rotarod Assay

Motor balance and coordination were measured on a rotarod (120 cm length; 3 cm diameter) at a constant speed of 20 rpm. Five sessions before the experiment, rats were taught, and only rats that were able to stay on the revolving rod for 5 min were involved. The rotarod test was done after the cylinder test. The time taken for each rat to fall off of a revolving rod (latency to fall) was documented as previously described by Sayed et al. [85].

#### 4.4.5. Animal Sacrificing and Midbrain Tissue Separation

At the conclusion of the experimental protocol on day 37, deep anesthesia was induced by intraperitoneal administration of sodium thiopental (50 mg/kg), followed by humane euthanasia. The brains were promptly harvested and transferred to a prechilled dissection platform to preserve tissue integrity. Six brains from each group were allocated for histological and immunohistochemical assessments and preserved in 10% neutral-buffered formalin. From the remaining specimens, the midbrain was carefully dissected and were homogenized in ice-cold PBS, PH 7.4, to produce a 10% homogenate and stored at −80 °C pending biochemical evaluation. Each parameter was assessed in 6 distinct rats for each group, and each sample was measured in technical triplicate.

### 4.5. Detection of Midbrain α-Synuclein and Dopamine

Alpha-synuclein and dopamine content in the midbrain tissue were evaluated by ELISA kit (Catalogue No: NBP-08175, Novus Biologicals, Centennial, CO, USA; CSB-E08660r, CUSABIO, Wuhan, China, respectively). Every step was executed aligned with the manufacturer’s criteria. The assay ranges for α-synuclein and dopamine are 15.63–1000 pg/mL, 0.156 ng/mL–10 ng/mL, respectively.

### 4.6. Measurement of Oxidative Stress Parameters in the Midbrain Homogenate

The midbrain tissue concentration of MDA, a result of lipid peroxidation, was determined using the FineTest colorimetric kit (Catalogue No: K084, Wuhan, China). The enzymatic antioxidant enzyme SOD activity was determined by employing the BioVision colorimetric kit (Catalogue No: K335-100, Milpitas, CA, USA). The homogenate content of reduced glutathione (GSH) was tested using a FineTest colorimetric kit (Catalogue No: EU2640, Wuhan, China); assay range is 1.56–100 µmole/L.

### 4.7. Inflammatory Hallmark Measurement in Midbrain Homogenate

ELISA kits were employed to quantify the tissue levels of tumor necrosis factor-α (TNF-α) (Catalogue No: E076Ra, BT LAB, Shanghai, China), interleukin-1β (IL-1β) (Catalogue No: E0119Ra, BT LAB, Shanghai, China), and interleukin-6 (IL-6) (Catalogue No: SEA079Ra, CloudClone Corp., Katy, TX, USA). All protocols were followed in consensus with the manufacturer’s specifications. The assay ranges for TNF-α, IL-1β, and IL-6 are 5–1000 ng/L, 0.2–60 ng/mL, and 7.8–500 pg/mL.

### 4.8. Assessment of PGC-1α, p-CREB, SIRT-1

To assess the amount of Phospho-cyclic AMP response element-binding protein (p-CREB) and Peroxisome proliferator-activated receptor gamma coactivator 1-alpha (PGC-1α). ELISA kits were utilized. These kits were obtained from SUNLONG, Zhejiang Province, China, (Catalogue No: SL1344Ra, SEA079Ra, respectively). The sirtuin 1 (SIRT-1) content in midbrain tissue was detected using an ELISA kit, which was purchased from BIOSS ANTIBODIES, Woburn, MA, USA. (Catalogue No: BSKR65911). Every step was executed in accordance with the manufacturer’s criteria. The detection ranges for PGC-1α, SIRT-1, and p-CREB are 16–500 pg/mL, 0.312–20 ng/mL, and 1–70 pg/mL.

### 4.9. Evaluation of Markers for Autophagy

By following the directions provided by the makers, midbrain of Beclin-1, ATG-7, and microtubules associated protein 1 light chain (LC3II) were measured using ELISA kits that are available for purchase. Fine Test’s ELISA kit was used to test Beclin-1 (Catalogue No: ER0416; Wuhan, China). ELK Biotechnology manufactured a kit that was used to measure autophagy-related protein-7 (ATG-7) content in the sample (Catalogue No: ELK9204; Sugar Land, TX, USA). We used AFG Scientific kit to test LC3II concentration (Catalogue No: EK721185; Arlington Heights, IL, USA). p62 (SQSTM1) was assayed using SUNLONG Biotech Co., LTD’s ELISA kit (Catalogue No: SL1363Ra; Hangzhou, Zhejiang Province, China). The detection range for Beclin-1, LC3II, ATG-7, and p-62 are 78.125–5000 pg/mL, 0.156–10 ng/mL, 0.16–10 ng/mL, and 54–4000 pg/mL, respectively.

### 4.10. Western Blotting of p-AMPK, MFN-1, and Drp-1

To determine whether midbrain tissue expressed mitochondrial biogenesis-related proteins, such as p-AMPK, MFN-1, and Drp-1, a Western blot was run. To summarize, the Bradford Protein Assay Kit (Cat #SK3041, Bio Basic Inc., Markham, ON, Canada) was used to extract total protein. Proteins of similar concentrations were isolated using SDS-PAGE and then deposited onto PVDF membranes. The membranes were treated overnight at 4 °C with primary antibodies against phosphorylated-adenosine monophosphate-activated protein kinase (p-AMPK) (1:1000) (Cell Signaling TECHNOLOGY, Inc., Catalogue No: #2531; Danvers, MA, USA), mitofusin (MFN-1) (1:500) (Gene Tex, Catalogue No: GTX66598; Clemensänger-Ost 9, Freising, Germany) and Drp-1 (1:2000) (Proteintech, Catalogue No: 12957-1-AP; Rosemont, IL, USA). After blocking with 3% BSA for 1 h at room temperature, the membranes were rinsed. Protein bands were exposed using an enhanced chemiluminescence (ECL) method after incubation with secondary antibodies conjugated with HRP. A loading control known as β-actin was used to normalize the band intensities, which were then quantified using densitometry.

### 4.11. Quantitative Reverse Transcription Polymerase Chain Reaction (QRT-PCR) for circDLGAP4, miR-34a-5p, and miR-134-5p Detection

The expression profiles of circDLGAP4, miR-34-5p, and miR-134-5p were evaluated in midbrain homogenates using RT–qPCR. Total RNA was purified with the SV Total RNA Isolation System (Promega, Madison, WI, USA), and 0.5–2 µg of the isolated RNA was subsequently converted into complementary DNA through the High-Capacity cDNA Reverse Transcription Kit (Catalogue No. K1621; Fermentas, Hanover, MD, USA). Amplification and real-time fluorescence detection were conducted using a StepOne™ Real-Time PCR System (Applied Biosystems, Waltham, MA, USA) with software version 3.1. The thermal conditions were optimized separately for each primer pair, particularly with respect to the required annealing temperature, Table 3.

### 4.12. Histopathological Studies

Brain specimens were gently rinsed and immersion-fixed in 10% neutral-buffered formalin for 72 h. The fixed tissues were then trimmed to the required dimensions, dehydrated through a graded series of ethanol, cleared in xylene, and impregnated with molten paraffin wax before being embedded in Paraplast medium. Serial sections measuring 5 µm in thickness were prepared using a rotary microtome, transferred onto glass slides, and stained with hematoxylin and eosin for routine evaluation of tissue architecture and histomorphological alterations [86].

#### 4.12.1. Immunohistochemical Staining of Brain Tissues for the Assessment of Tyrosine Hydroxylase

Deparaffinized brain slices (5 µm) were inoculated with 3% H_2_O_2_ for 20 min and then incubated overnight with anti-TH antibody (Catalogue No.: PA1-4679; Invitrogen, Thermo Fisher Scientific, Waltham, MA, USA; 1:500 dilution). Sections were washed with PBS, submerged with HRP-conjugated EnVision antibody for 20 min, developed with DAB for 10 min, counterstained with hematoxylin, cleared out, and cover-slipped for microscopic assessment.

#### 4.12.2. Microanalysis

The examiner histologist randomly chose a minimum of six non-overlapping fields per tissue section of each sample at ×40 magnification, applying a Leica Microsystems GmbH Full HD microscopic imaging system with Leica Application software. The scanned Hematoxylin and Eosin-stained sections were used to calculate the % of degenerated neurons per total neuronal cells within 1 mm^2^ according to Mousa et al. [87]. The area percentage of tyrosine hydroxylase immunohistochemical expression levels was calculated in the immunostained sections [88].

### 4.13. Molecular Docking

The potential binding modes of the Mexil with CREBBP [71] and α-synuclein [89] using PDB IDs 3P1C and 7WMM were investigated using molecular docking [90,91]. All ligands were reprocessed before docking. The preparation procedure included generation of 3d ligand conformations, assignment of formal charges, enumeration of relevant protonation states, addition of hydrogens, correction of aromaticity and valence, assignment of partial atomic charges, energy minimization, and removal of chemically implausible tautomeric states.

For protonatable amines, the expected predominant species at approximately physiological pH was used. In particular, primary amines were prepared predominantly in their protonated ammonium form, and nonionizable functional groups were retained in their neutral forms.

Docking simulations were performed with AutoDock Vina v1.2.7 [92], while ligand and protein preparation (conversion to pdbqt format) was carried out using OpenBabel [93]. Visualization and interaction analyses were conducted in Discovery Studio [94]. AutoDock Vina applies a united atom scoring function with an Amber force field, integrating a gradient-based local search genetic algorithm with global optimization to predict ligand binding conformations. The active sites of CREBBP and α-synuclein were defined based on their co-crystallized ligands (PDB IDs: ALY and 1KI) using a grid box dimension of 20 × 20 × 20 Å along the x, y, and z axes. Binding affinities and interaction profiles were then evaluated through docking scores and 2D interaction diagrams.

### 4.14. Statistical Data Analysis

Data were reported as mean ± standard deviation (SD). GraphPad Prism version 8 was implemented for statistical computations and graphing. Before inferential analysis, conformity to a normal distribution was examined by applying the Shapiro–Wilk test. Statistical differences between groups were established utilising one-way ANOVA with Tukey’s post hoc multiple comparison test, with *p*-values < 0.05 deemed significant.

### 4.15. Limitations of the Work

Notwithstanding these encouraging findings, several limitations merit acknowledgment before clinical relevance can be established. Although the present study demonstrates coordinated modulation across the oxidative stress–circDLGAP4/miR-134-5p/p-CREB axis, mitochondrial biogenesis and dynamics, autophagy, and neuroinflammatory signaling, direct causal relationships among these interconnected pathways were not established, as this would necessitate genetic knockdown or pharmacological inhibition approaches beyond the scope of the present investigation. Furthermore, ultrastructural mitochondrial alterations characteristic of mitochondrial dysfunction was not assessed in this study. Future investigations incorporating targeted pathway inhibition strategies, alongside transmission electron microscopy, will be essential to establish mechanistic causality and to provide structural corroboration of the biochemical alterations reported herein. Also, this study shows the lack of direct measurement of cellular uptake and intracellular localization of the optimized niosomal formulation. However, the observed biological effects provide indirect evidence in favor of the activity of the optimized niosomal formulation but do not directly confirm cellular internalization. The cellular uptake and intracellular localization should be studied in the future by suitable quantitative methods. The absence of free Mexil and blank niosome controls prevents a definite conclusion on whether the effects seen are due to the drug or the niosomal delivery system. In addition, the lack of comparative pharmacokinetic, biodistribution, and brain penetration data prevents direct assessment of formulation-specific delivery advantages and more comprehensive physicochemical parameters. Further work with these complementary approaches is needed to delineate the contribution and therapeutic value of niosomal delivery. Moreover, the present formulation-development strategy is the absence of a direct comparative in vivo evaluation of N4 against the other formulations. Furthermore, future studies employing cell-specific approaches would provide more definitive evidence regarding the localization of these molecular responses to dopaminergic neurons.

## 5. Conclusions

Overall, our results suggest that niosomal Mexil exerts robust neuroprotective effects against rotenone-induced Parkinsonism-like neurodegeneration by acting on the co-regulation of oxidative stress, inflammation, mitochondrial dysfunction, autophagy, and apoptosis. The neuroprotective effects of niosomal Mexil seem to be tightly associated with the upregulation of circDLGAP4/miR-134-5p/p-CREB signaling, along with increased expression of p-AMPK, PGC-1α, and SIRT-1, as well as elevated cellular ATP content, which appear to restore mitochondrial dynamics and autophagic capacity while suppressing apoptotic activity. Together, these results support the neuroprotective potential of niosomal Mexil in the present rotenone-induced model of Parkinsonian neurodegeneration, with its effects related to the coordinated modulation of interconnected oxidative, inflammatory, and mitochondrial pathways rather than a single mechanistic target. While these results provide a rationale for further investigation, the therapeutic relevance of niosomal Mexil in Parkinson’s disease needs to be validated in complementary experimental models and more comprehensive translational studies. Further future studies are therefore warranted to establish the reproducibility, mechanistic contribution, and possible therapeutic applicability of this formulation.

## Figures and Tables

**Table 1:** Results of molecular docking of the crystallized ligand, standard drug and mexiletine in conjunction with CREBBP (PDB ID: 3P1C), and α-synuclein (PDB ID: 7WMM).

Target Ligand Complex	Docking Scores (kcal/mol)	RMSD (Å)	Interacting Residues
Crystallized molecules (PBD ID: ALY) + CREBBP	−4.74	1.25	Arg1173, Asn1168, Val1174, and Leu1120
Mexiletine + CREBBP	−5.2	1.59	Val1174 and Leu1120, Asn1168 and Ile1122
Crystallized molecules (PBD ID: 1KI) + α-synuclein	−6.425	1.26	Glu83, Lys80, Val82, and Tyr39
Mexiletine + α-synuclein	−4.9	1.45	Glu46 and Tyr39

CREBBP: CREB-binding protein, PBD: Protein Data Bank, Arg1173: Arginine 1173, Asn1168: Asparagine 1168, Val1174: Valine 1174, Leu1120: Leucine 1120, Ile1122: Isoleucine 1122, Glu83: Glutamic Acid 83, Glu46: Glutamic Acid 46, Lys80: Lysine 80, Val82: Valine 82, Tyr39: Tyrosine 39.

**Table 2:** Amounts of components of the prepared Mexil-loaded niosomal dispersions.

Formula Code	Amount (mg)			
	Mexil	Span 60	CHL	LEC
N1	50	75	75	150
N2	50	50	100	150
N3	50	100	50	150
N4	50	75	75	225
N5	50	50	100	225
N6	50	100	50	225

Mexil: mexiletine; CHL: cholesterol; LEC: lecithin.

**Table 3:** Primer sequences.

	Forward Sequence	Reverse Sequence
miR-34a-5p	CGCGTGGCAGTGTCTTAGCT Accession No. MIMAT0000815	AGTGCAGGGTCCGAGGTATT
miR-134-5p	TGTGACTGGTTGACCAGAGGG Accession No. MIMAT0000840	AGTGCAGGGTCCGAGGTATT
U6	GCATCTGAGTCGCCACTGTA Accession No. XR_007492463.1	CGCTTCACGAATTTGCGTGTCAT
circDLGAP4	CTCCCGCAGCCGCATC Accession No. XM_063283180.1	AGAGGGGAGGCACGGG
GAPDH	TTCAACAGCAACTCCCATT Accession No. NM_017008.4	CACCACCCTGTTGCTGTA

## Data Availability

Data is contained within the article.

## Abbreviations

The following abbreviations are used in this manuscript:

ALY	N^6^-acetyl-L-lysine (co-crystallized ligand code, PDB 3P1C)
AMPK	AMP-activated protein kinase
ANOVA	analysis of variance
ARRIVE	Animal Research: Reporting of In Vivo Experiments
ATG	autophagy-related protein (ATG5, ATG7/ATG-7, ATG10, ATG12)
ATP	adenosine triphosphate
BBB	blood–brain barrier
Bcl-2	B-cell lymphoma 2
BDNF	brain-derived neurotrophic factor
BSA	bovine serum albumin
CaMKII	Ca^2+^/calmodulin-dependent protein kinase II
cAMP	cyclic adenosine monophosphate
CBP	CREB-binding protein
Cdna	complementary DNA
CHL	Cholesterol
circDLGAP4	circular RNA derived from the discs large-associated protein 4 (DLGAP4) gene
circRNA	circular RNA
CREB	cAMP response element-binding protein
CREBBP	CREB-binding protein (gene/protein symbol)
DAB	3,3′-diaminobenzidine
Drp-1	dynamin-related protein 1
ECL	enhanced chemiluminescence
EE%	entrapment efficiency (percentage)
ELISA	enzyme-linked immunosorbent assay
FTIR	Fourier transform infrared (spectroscopy)
GAPDH	glyceraldehyde-3-phosphate dehydrogenase
GSH	reduced glutathione
HCl	Hydrochloride
HRP	horseradish peroxidase
IACUC	Institutional Animal Care and Use Committee
IL-1β	interleukin-1 beta
IL-6	interleukin-6
KAT3A	lysine acetyltransferase 3A
LC3-II	microtubule-associated protein 1 light chain 3-II (lipidated form of LC3-I)
LEC	Lecithin
MDA	Malondialdehyde
Mexil	Mexiletine
MFN-1	mitofusin-1 (also written MNF in the Methods)
miR-134-5p	microRNA-134-5p
miR-34a-5p	microRNA-34a-5p
MiRNA	microRNA
MPP^+^	1-methyl-4-phenylpyridinium
MPTP	1-methyl-4-phenyl-1,2,3,6-tetrahydropyridine
MtDNA	mitochondrial DNA
mTORC1	mechanistic target of rapamycin complex 1
MWCO	molecular weight cut-off
NAD+	nicotinamide adenine dinucleotide (oxidized form)
NcRNA	noncoding RNA
NRF1	nuclear respiratory factor 1
p-AMPK	phosphorylated AMP-activated protein kinase
p-CREB	phosphorylated cAMP response element-binding protein
p.o.	per os (oral administration)
p62/SQSTM1	sequestosome 1
PBS	phosphate-buffered saline
PD	Parkinson’s disease
PDB	Protein Data Bank
PDI	polydispersity index
PGC-1α	peroxisome proliferator-activated receptor gamma coactivator 1-alpha
PI3-kinase	phosphoinositide 3-kinase
PS	particle size (written “PZ” in the Stability Study section)
PTEN	phosphatase and tensin homolog
PVDF	polyvinylidene difluoride
RMSD	root-mean-square deviation
ROS	reactive oxygen species
Rot	Rotenone
RT–qPCR	reverse transcription–quantitative polymerase chain reaction
s.c.	Subcutaneous
SD	standard deviation
SDS-PAGE	sodium dodecyl sulfate–polyacrylamide gel electrophoresis
SF	striatal fibers (also labelled ST)
SIRT-1	sirtuin 1
SN	substantia nigra
SNpc	substantia nigra pars compacta
SOD	superoxide dismutase
Span 60	sorbitan monostearate
Tc	phase-transition temperature
TFAM	mitochondrial transcription factor A
TFEB	transcription factor EB
TH	tyrosine hydroxylase
TNF-α	tumor necrosis factor alpha
ZP	zeta potential
